# Fine-needle percutaneous muscle microbiopsy technique as a feasible tool to address histological analysis in young children with cerebral palsy and age-matched typically developing children

**DOI:** 10.1371/journal.pone.0294395

**Published:** 2023-11-22

**Authors:** Jorieke Deschrevel, Karen Maes, Anke Andries, Nathalie De Beukelaer, Marlies Corvelyn, Domiziana Costamagna, Anja Van Campenhout, Eva De Wachter, Kaat Desloovere, Anouk Agten, Frank Vandenabeele, Stefaan Nijs, Ghislaine Gayan-Ramirez

**Affiliations:** 1 Department of Chronic Diseases and Metabolism, Laboratory of Respiratory Diseases and Thoracic Surgery, KU Leuven, Leuven, Belgium; 2 Department of Rehabilitation Sciences, Research Group for Neurorehabilitation, KU Leuven, Leuven, Belgium; 3 Department of Development and Regeneration, Stem Cell Biology and Embryology Unit, KU Leuven, Leuven, Belgium; 4 Department of Development and Regeneration, KU Leuven, Leuven, Belgium; 5 Department of Orthopaedic Surgery, University Hospitals Leuven, Leuven, Belgium; 6 Faculty of Rehabilitation Sciences, Rehabilitation Research Center, Hasselt University, Diepenbeek, Belgium; 7 Department of Trauma Surgery, University Hospitals Leuven, Leuven, Belgium; Maastricht University Faculty of Health, Medicine and Life Sciences: Maastricht Universitair Medisch Centrum+, NETHERLANDS

## Abstract

Cerebral palsy (CP) is a heterogeneous group of motor disorders attributed to a non-progressive lesion in the developing brain. Knowledge on skeletal muscle properties is important to understand the impact of CP and treatment but data at the microscopic levels are limited and inconsistent. Currently, muscle biopsies are collected during surgery and are restricted to CP eligible for such treatment or they may refer to another muscle or older children in typically developing (TD) biopsies. A minimally invasive technique to collect (repeated) muscle biopsies in young CP and TD children is needed to provide insights into the early muscle microscopic alterations and their evolution in CP. This paper describes the protocol used to 1) collect microbiopsies of the medial gastrocnemius (MG) and semitendinosus (ST) in CP children and age-matched TD children, 2) handle the biopsies for histology, 3) stain the biopsies to address muscle structure (Hematoxylin & Eosin), fiber size and proportion (myosin heavy chain), counting of the satellite cells (Pax7) and capillaries (CD31). Technique feasibility and safety as well as staining feasibility and measure accuracy were evaluated. Two microbiopsies per muscle were collected in 56 CP (5.8±1.1 yr) and 32 TD (6±1.1 yr) children using ultrasound-guided percutaneous microbiopsy technique. The biopsy procedure was safe (absence of complications) and well tolerated (Score pain using Wong-Baker faces). Cross-sectionally orientated fibers were found in 86% (CP) and 92% (TD) of the biopsies with 60% (CP) and 85% (TD) containing more than 150 fibers. Fiber staining was successful in all MG biopsies but failed in 30% (CP) and 16% (TD) of the ST biopsies. Satellite cell staining was successful in 89% (CP) and 85% (TD) for MG and in 70% (CP) and 90% (TD) for ST biopsies, while capillary staining was successful in 88% (CP) and 100% (TD) of the MG and in 86% (CP) and 90% (TD) for the ST biopsies. Intraclass coefficient correlation showed reliable and reproducible measures of all outcomes. This study shows that the percutaneous microbiopsy technique is a safe and feasible tool to collect (repeated) muscle biopsies in young CP and TD children for histological analysis and it provides sufficient muscle tissue of good quality for reliable quantification.

## Introduction

Cerebral palsy (CP) is a heterogeneous group of motor disorders attributed to a non-progressive lesion in the developing fetal or infant brain occurring in 1.5 out of 1000 live births [1]. CP is firstly characterized by neural deficits and secondly by impairments in the musculoskeletal system which increase with age [2]. The clinical symptoms of the neuromuscular impairment include spasticity and increased muscle stiffness, muscle weakness, loss of selectivity and decreased functional ability limiting physical activity and participation to social activities [2–4]. The gross motor function classification system (GMFCS) is the golden standard in classifying the functional ability of children with CP where mobility is scored on a scale from I (less disability) to V (severe dysfunction) [2].

Knowledge on skeletal muscle properties is essential to understand how CP affects the muscles and how these alterations are eventually counterbalanced, minimized or possibly aggravated by treatment. So far, multiple imaging techniques such as magnetic resonance imaging and ultrasound have been used for visualizing the macroscopic muscle properties [5]. These techniques have provided consistent evidence for shorter muscle, reduced muscle volume, muscle cross-sectional area, and thickness, and increased echogenicity intensity in muscle of children with CP compared to typically developing (TD) children [3,5–7]. They are also useful to follow disease evolution and to document the potential benefit of the treatment therapy on a macroscopic level. Nevertheless, these techniques do not provide information about the microscopic muscle properties (e.g. fiber dimension and proportion, amount of capillary and satellite cells) for which a muscle biopsy is required.

There are several ways to collect muscle biopsies in humans, either with an open muscle biopsy during surgery or with a semi-open muscle biopsy using the percutaneous needle technique. An open muscle biopsy provides a large amount of tissue but is rather invasive as it needs an incision through the skin and fascia of about 4–6 cm [8]. The semi-open muscle biopsy techniques use either a percutaneous needle to collect the biopsy such as the Bergström needle or a Tilley-Henckel punch forceps for the percutaneous conchotome muscle biopsy technique. Semi-open muscle biopsies are less invasive although they still require an incision of 5–10 mm through the skin and fascia due to the relatively large needle size (diameter of 3.5 mm) [9–11]. This can be painful, cause scar tissue and is not always well tolerated by patients. Using these approaches, several alterations at the microscopic level have been reported in the muscle of children with CP [12–25]. However, inconsistent data have been reported regarding fiber size [2,7,17], predominance of a given fiber type [2], amount of satellite cells [17,19,21,26] and capillaries [20]. To our knowledge, there is furthermore no longitudinal study analyzing the evolution of muscle alterations at the microscopic level in young children with CP to address the early disease stage and to follow up how these muscle alterations evolve during the child’s development. Obviously, to improve the weaknesses of the previous studies on microscopic muscle properties, there is a need for a suitable biopsy technique which on the one hand is minimally invasive to allow acquiring (repeated) muscle biopsies in (very) young children with CP and on the other hand provides sufficient material to allow appropriate tissue fixation and subsequent histological analysis. Additionally, this technique should also be compatible with collecting muscle biopsies in TD children for optimal comparison with age-matched children using the same muscle as in children with CP.

The current methodological paper describes a new approach used for the standardized collection of muscle microbiopsies in children with CP and age-matched TD children and the subsequent fixation and (immuno-)staining protocol for downstream analysis of muscle pathology. We chose the fine needle microbiopsy technique also termed percutaneous microbiopsy technique, which is less invasive and has a needle diameter more suitable for collecting biopsies in young children. Previous studies in adults have shown that this technique was well tolerated and provided sufficient muscle tissue of good quality to perform histological analysis [11,27,28]. These points were systematically addressed in young children in the current paper. We adapted the percutaneous microbiopsy technique previously described in adults [27,28] to collect and handle microbiopsies of the Medial Gastrocnemius (MG) and Semitendinosus (ST) of young children for histological analysis and documented both the feasibility and safety of using such a technique in such age group.

## Methods

### Study population and targeted muscles

In this study, 56 children with CP and 32 TD children with a range of 2 to 9 years (yr) old were included. For the children with CP, children with GMFCS level I, II or III and with uni- or bilateral involvement were eligible for inclusion. Children with CP were excluded in case of presence of dystonia or ataxia, botulinum toxin-A (BoNT-A) injections within the last 6 months (m), orthopedic surgery less than 2 yr ago as well as any muscle surgery on the included muscles or with severe co-morbidities. All patients received usual care including regular physical therapy (1 to 5 sessions per week, with an average duration of 43 minutes (min) per week) and most patients used ankle foot orthoses during the day and fixed ankle foot orthoses, often combined with knee extension braces, during the night as standard of care. TD children with no history of neurological or musculoskeletal disorders were included, while those practicing sport activities for more than 3 hours (h) per week were excluded. For children with CP, the muscles of the most affected side according to their medical record were assessed, while for TD children the target side was randomly defined. Children with CP were recruited through the Cerebral Palsy Reference Center of the University Hospitals Leuven (Leuven, Belgium) and the Clinical Motion Analysis Laboratory at the University Hospitals Leuven (Pellenberg, Belgium). TD children were recruited from the Traumatology Unit or the Ear-Nose-Throat Unit of the University Hospitals Leuven (Belgium). Parents and children were contacted by the researchers either via face-to-face conversation at the clinical appointment, or via e-mail or by phone with the permission of the supervising clinician. Ethical approval for this study was provided by the Ethical Committee UZ Leuven/KU Leuven (S61110 and S62645). The written informed consent was provided and signed by the parents or guardians of the children prior to the biopsy collection.

Ultrasonography-guided percutaneous microbiopsies were collected from the MG and ST. In children with CP, the biopsies were taken during procedures that required either general anesthesia namely during BoNT-A injections or orthopedic surgery or by sedating the child with nitrous oxide and local anesthesia to collect a follow-up biopsy after treatment. Biopsies from TD children were only collected under general anesthesia during removal of orthopedic implants of the upper limbs or during Ear-Nose-Throat surgery.

The biopsies were taken by an orthopedic surgeon with experience in muscle biopsy sampling and expertise in neuro-orthopedic disorders such as CP. All procedures took place at the University Hospitals of Leuven (Leuven, Belgium).

### Microbiopsy device description and functioning

**Fig 1:**
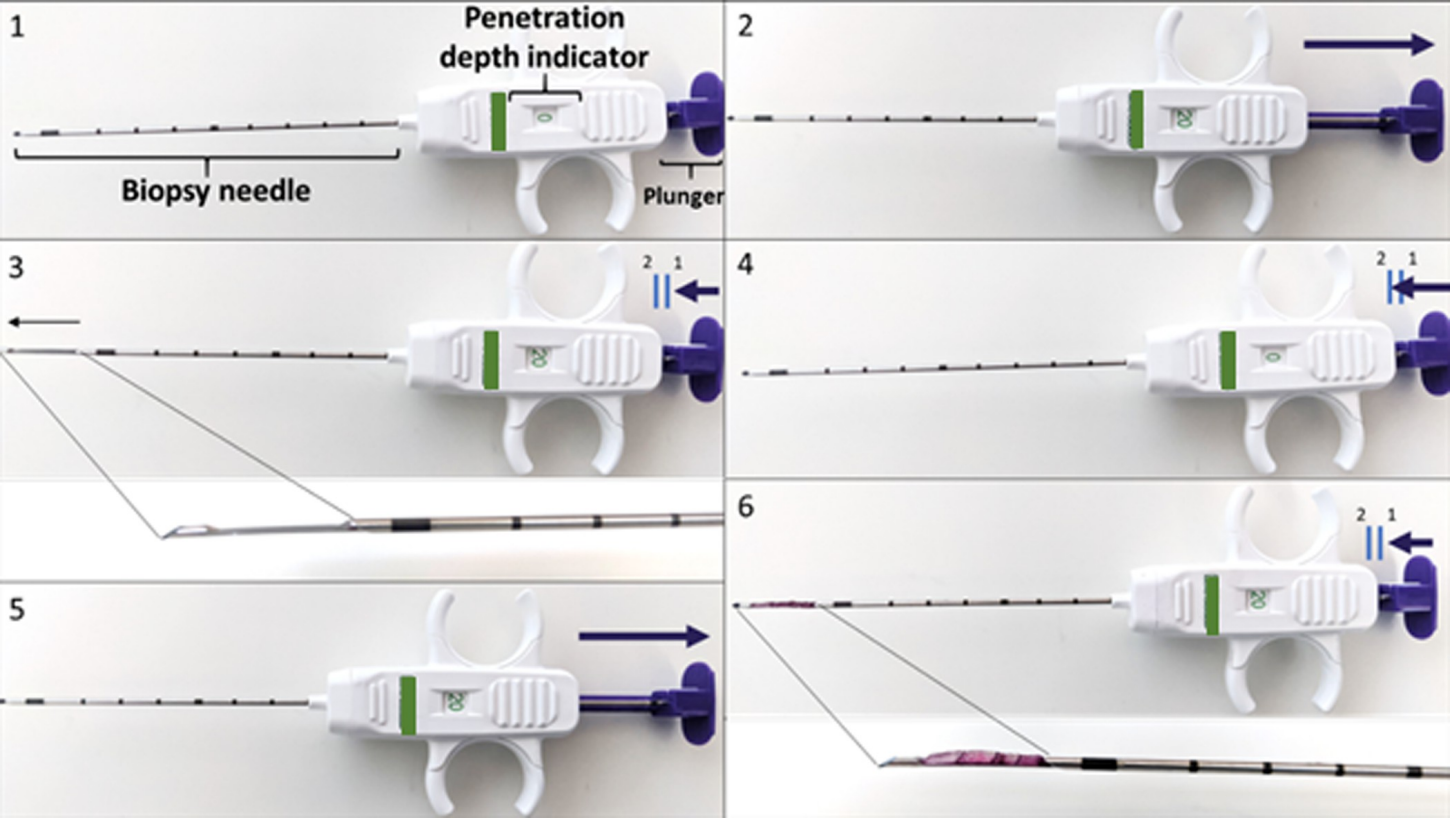
Illustrations of the different steps to handle the microbiopsy needle for collecting muscle tissue. 1) overview of the microbiopsy needle with its different parts; 2) Pulling of the plunger till the desired penetration depth as shown on the indicator; 3) After entrance of the needle into the tissue, the plunger is pushed down until the first stop, pushing out the notch of the needle; 4) The plunger is then pushed down until the second stop to fire the cutting area; 5–6) To release the biopsy, the plunger is pulled up and then pushed until the first stop.

The microbiopsy device consists of a disposable core biopsy instrument with a 10 cm long 16 Gauge (G) needle with an outer diameter of 1.651 ± 0.0005 mm (Bard Mission™ Disposable Core Biopsy Instrument 16G x 10 cm-Semi-Automatic) equipped with a semi-automatic firing system and a penetration depth indicator for 10- or 20-mm. Needle penetration depth was pre-set at 20 mm in this study. After pulling the plunger, the needle was inserted into the tissue under ultrasound guidance. The plunger was then pushed till the first stop, causing the notch of the needle to be pushed out. Because the chosen needles have a good visibility on ultrasound, the position of the needle was followed and checked with ultrasound as detailed later, before pressing the plunger to fire the cutting area. Once the needle was removed out of the tissue, the plunger was first pulled and then pushed till the first stop to expose the microbiopsy muscle sample (Fig 1).

### Muscle microbiopsy procedure

**Fig 2:**
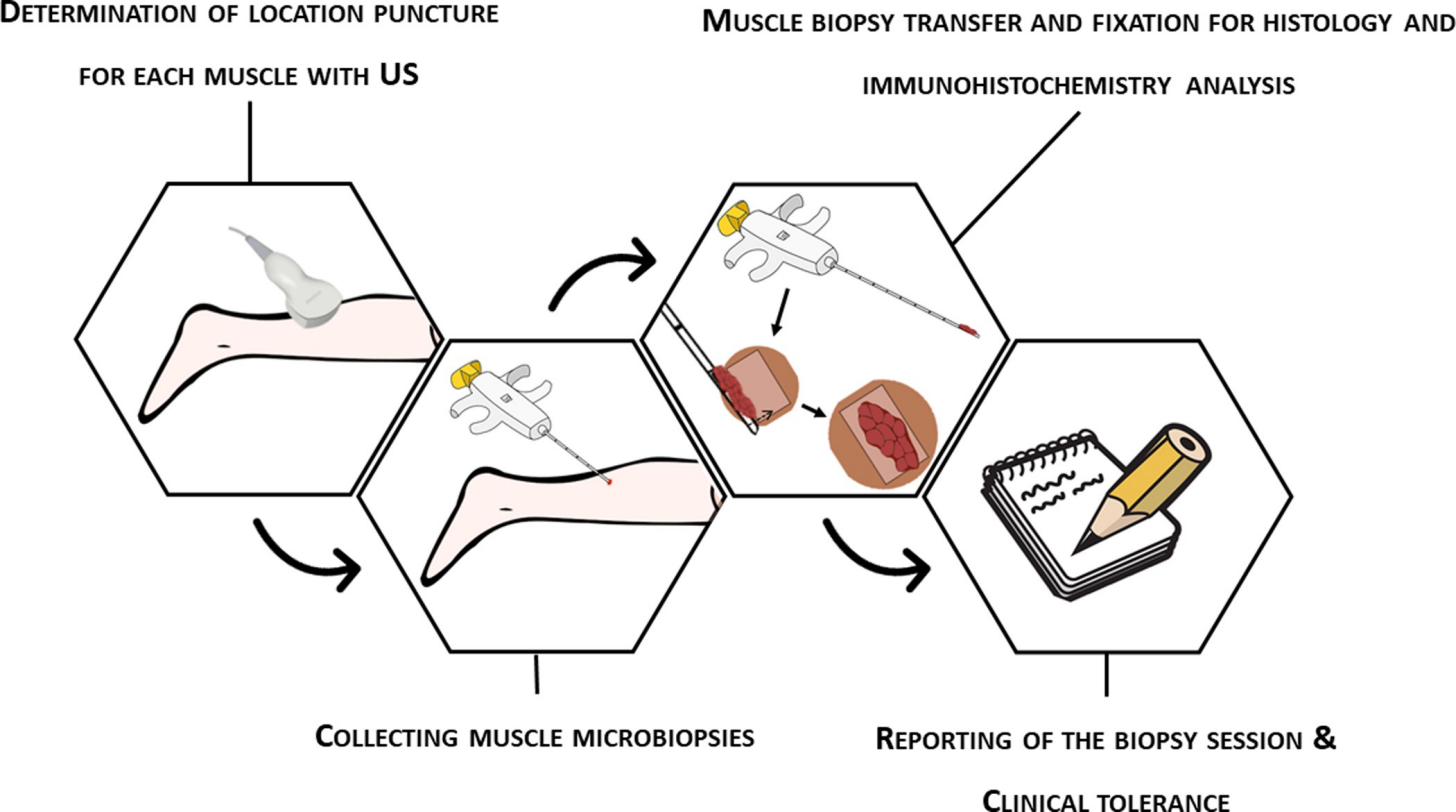
Schematic overview of the different steps of the muscle microbiopsy procedure. The first step is to collect the muscle microbiopsy, followed by the transfer of the muscle sample for histological analysis and lastly, report the biopsy session. See text for details.

The different steps to collect the muscle microbiopsy samples and transfer it for histological analysis are depicted in Fig 2 and detailed thereafter.

### Collecting muscle microbiopsies

**Fig 3:**
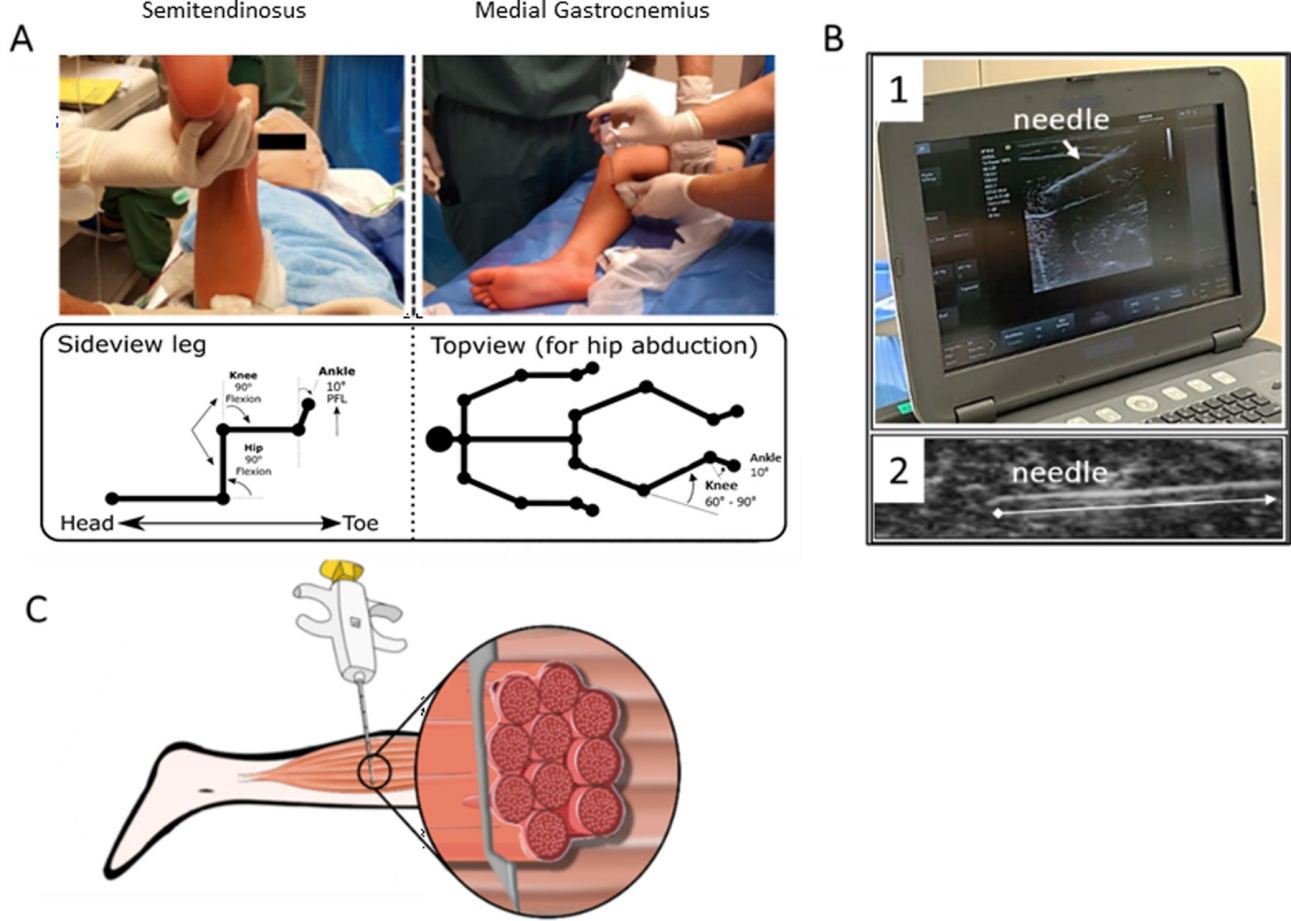
Detailed overview on how the muscle microbiopsies were collected. A) overview of the position of the leg when collecting the microbiopsy of the Medial Gastrocnemius (left) and the Semitendinosus (right); B) Ultrasound image showing the position of the microbiopsy needle (white arrow) through the muscle (B.1) and close-up view of the needle into the muscle (B.2); C) Schematic image representing the way the opening tip of the needle biopsy is inserted into the muscle to obtain a perpendicular position of the needle relative to the orientation of the muscle fibers.

Once fully anesthetized, hip, knee and ankle joint configurations of the leg were standardized to gain access to the muscles which allowed to perform ultrasound guidance for obtaining muscle biopsies under standardized muscle conditions. The child was in a supine position while biopsies were taken. To collect the biopsy of the ST muscle, the leg was positioned with the hip and knee flexed to 90°, as if you would assess the popliteal angle (Fig 3A right panel). To collect the MG biopsies, the leg was positioned with a slightly abducted and flexed hip, the knee in 60° to 90° flexion and the ankle in resting position without external manipulation to assure the ankle degree results on average at 10° of plantar flexion position (Fig 3A left panel).

After locating the mid-belly cross-sectional area of the targeted muscle with ultrasound guidance, a 16G coaxial biopsy needle was inserted through the skin and upper fascia of the muscle. The needle depth and position were followed under ultrasound guidance (Fig 3B). Care was taken to position the opening tip of the biopsy needle perpendicularly to the orientation of the muscle fibers to obtain a biopsy sample cross-sectionally orientated to the fibers (Fig 3C). In the next step, the plunger was pushed down to the first stop and the location of the sample notch was examined on the ultrasound to aim for the maximal length of the biopsy collection. The plunger was then pushed to fire the cutting area, the needle removed from the muscle and the muscle sample released by pulling the plunger back to expose the notch with the sampled tissue (Fig 1, steps 5 and 6). For each child, three to four muscle samples per muscle were collected using the same needle insertion point, but with a slightly different orientation. Two samples were handled for histology analysis as described thereafter while the other samples were used for other purposes not described in the current paper [26]. After the biopsy collection, pressure was immediately applied with a gauze on the site of the biopsy and ice pack cooling was applied to avoid hematoma. Pain medication was prescribed to be taken when needed: it consisted in paracetamol or ibuprofen depending on the type of additional procedure and/or comorbidities.

#### Muscle biopsy transfer and fixation for histology and immunohistochemistry analysis

**Fig 4:**
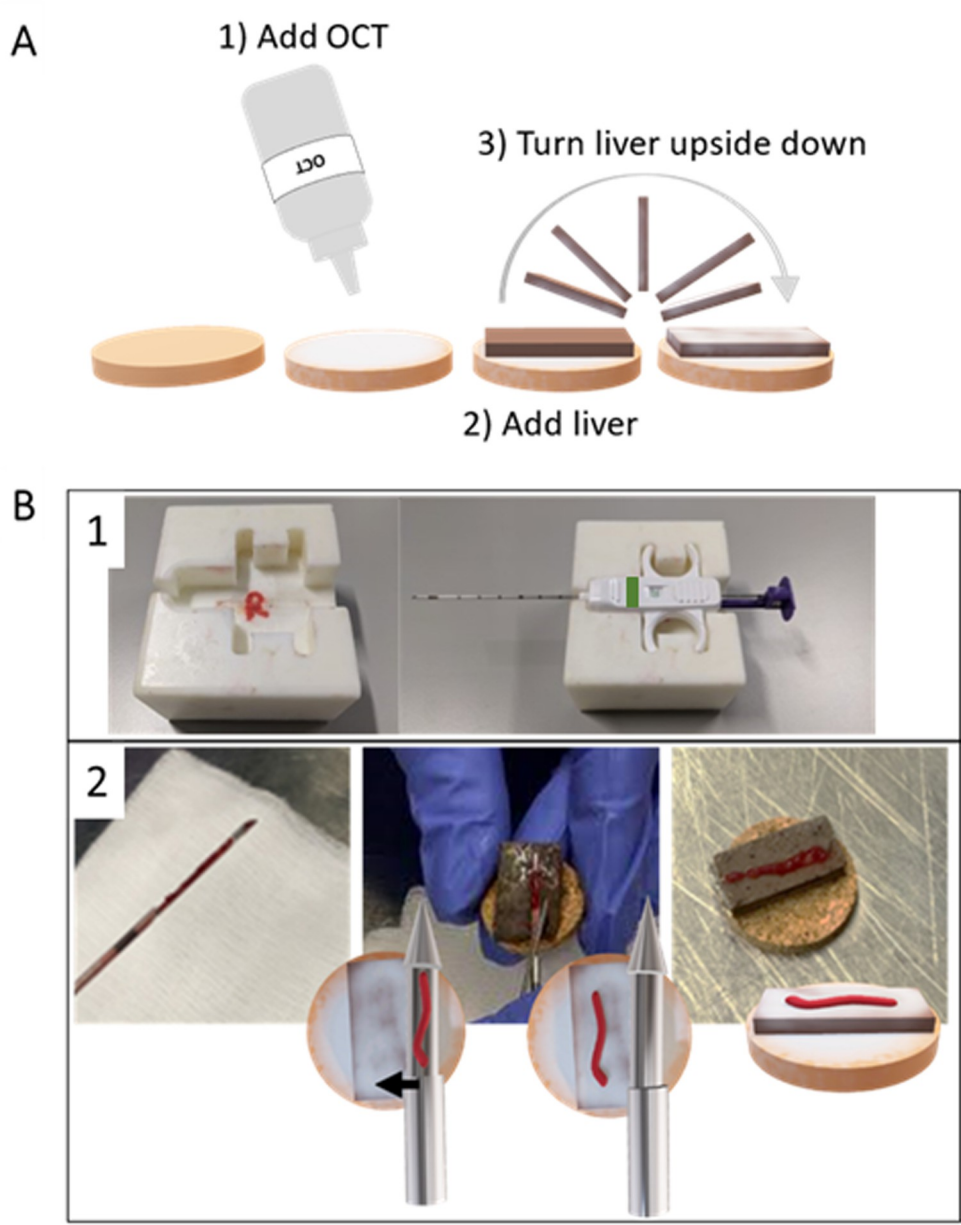
Overview of the muscle biopsy transfer and its fixation for subsequent analysis. A) Overview of the different steps to prepare the liver-cork support where the biopsy is placed on in next instance. First, OCT is put on the cork, then the piece of liver is dipped into the OCT so that the part on top is now covered with a very thin layer of OCT; B) Overview of the transfer of the muscle biopsy to the cork support with B.1 showing the home-made needle holder and B.2 the positioning of the cork under the biopsy needle. Of importance is to transfer the muscle sample to the cork with the help of a needle avoiding any changes to the muscle sample orientation in order to maintain the cross-sectional orientation of the muscle fibers.

The muscle biopsy was first transferred from the biopsy needle onto a piece of pig liver covered on both sides with a very thin layer of tissue-tek optimal cutting temperature (OCT) compound and placed on a cork (Fig 4A). The piece of liver was used to be able to cut the entire muscle biopsy without hitting the cork with the blade during sectioning. To avoid freezing artefacts, only a very thin layer of OCT between the biopsy and the piece of liver was used.

During the transfer phase (Fig 4B), careful attention was paid to transfer the muscle biopsy from the microbiopsy needle to its position in order to keep the cross-sectional orientation of the muscle fibers. To achieve this goal, the biopsy needle was fixed in a home-made needle holder (Fig 4B.1) while placing the cork support under the needle. Next, the muscle sample was gently pushed forward horizontally using a 18G syringe needle placed parallel to the muscle sample (Fig 4B.2). After transfer, the muscle biopsy on its support was immediately frozen for at least 30 seconds (s) in isopentane cooled with liquid nitrogen. The sample was placed in a tube and kept in liquid nitrogen until its storage at -80°C.

#### Reporting of the biopsy session

The entire course of the biopsy session was recorded with a voice recorder to make a biopsy report afterwards. For each subject, this information included subject information, position of the leg for ST and MG, location of the needle, visual description of the biopsy sample, information about the transfer and any other comments regarding the procedure. This information gave a general idea of the biopsy session and could help explaining potential problems.

#### Clinical tolerance

**Fig 5:**
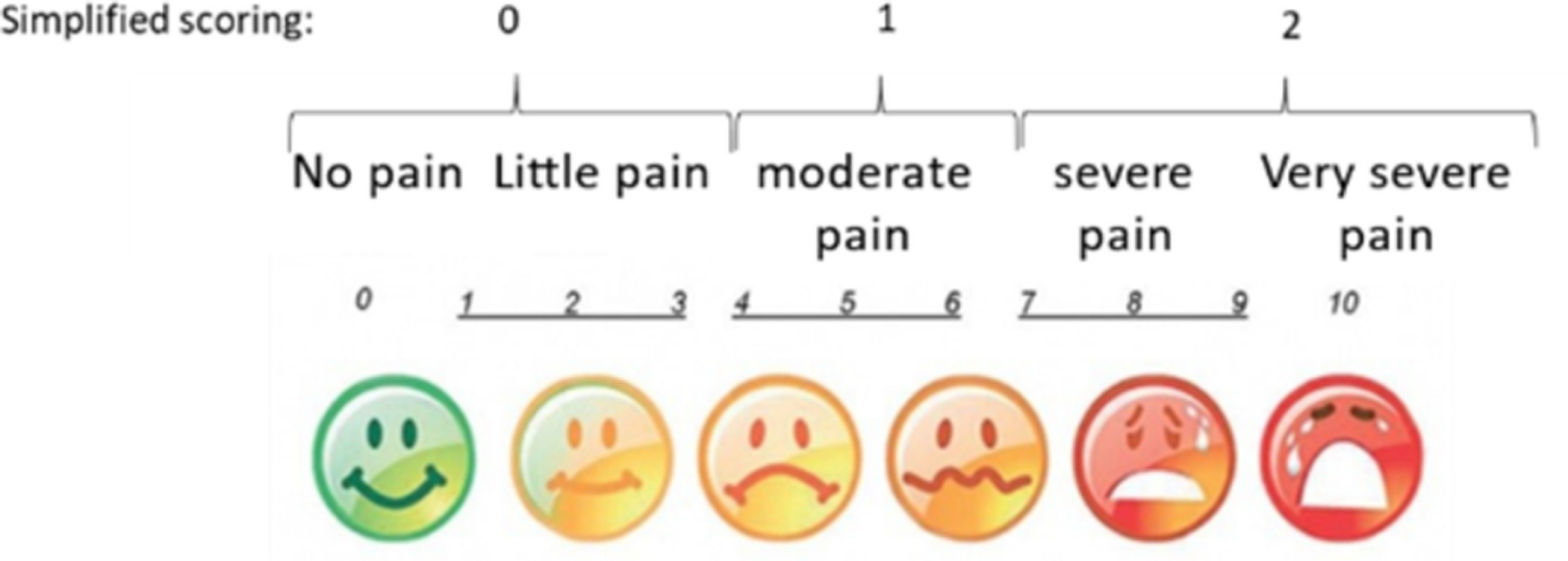
Evaluation of the clinical tolerance of the biopsy process. The Dutch version of the Wong-Baker Faces pain rating scale from www.pijn.nl was used to evaluate the pain one day and two days after the biopsy was taken. For convenience, this Dutch version was translated to English and the simplified scoring approach used for this study is indicated.

The tolerance of the biopsy process for the child was evaluated with support of the parents through a custom-made questionnaire. The pain score was indicated using the Wong–Baker Faces Pain Rating Scale at the day of the biopsy collection and 2 days after the biopsy (Fig 5) [29]. The pain score was then converted to a three-point system to facilitate the quantification with 0 = no to little pain (0–3 on pain scale), 1 = moderate pain (4–6 on pain scale) and 2 severe to very severe pain (7–10).

### Microbiopsy analysis

#### Sectioning of the frozen muscle sample using a cryostat

Sections of 5 μm were cut using a cryostat maintained at -20°C and each section was transferred on a charged slide (SuperFrost plus, VWR). Attention was paid to obtain sections of the entire muscle sample meaning that the first few sections were discarded. The biopsy was cut and air-dried for at least one hour before being stored at -20°C.

#### Muscle structure semi quantification: Hematoxylin & Eosin staining

The Hematoxylin & Eosin staining (H&E) staining protocol was based on the manufacturer’s protocol from the Mayer’s hemalum solution from Sigma-Aldrich. For each muscle, one section brought at room temperature was first rinsed in distilled water and then stained for one minute in filtered hematoxylin solution (1.09249, Mayer’s hemalum solution, Sigma-Aldrich). The section was then rinsed in 0.1% hydrochloric acid for 2 sec and in running tap water for 10 min. Subsequently, the section was stained with eosin (1.09844, eosin Y-solution, Sigma-Aldrich with 0.2% glacial acetic acid) for 1 min, washed in tap water till the water was clear and finally rinsed in distilled water. The section was then dehydrated by successive rinsing in distilled water, 50% ethanol (EtOH), 70% EtOH, 90% EtOH, 100% EtOH, 5 min in 1:1 xylene EtOH followed by 5 min in xylene. The stained muscle sample was covered with DPX mounting media (VWR) and a cover slip, and dried for 24 h before pictures were taken. The entire muscle biopsy was visualized under a brightfield microscope (Leica DMi8) at magnification x20, and qualitative evaluation of the section was performed to estimate the quantity of muscle tissue, cross-sectional orientation of the muscle fibers and presence of freezing artefacts.

#### Muscle fiber cross-sectional area (CSA) and proportion: myosin heavy chain (MHC) immunostaining

**Fig 6:**
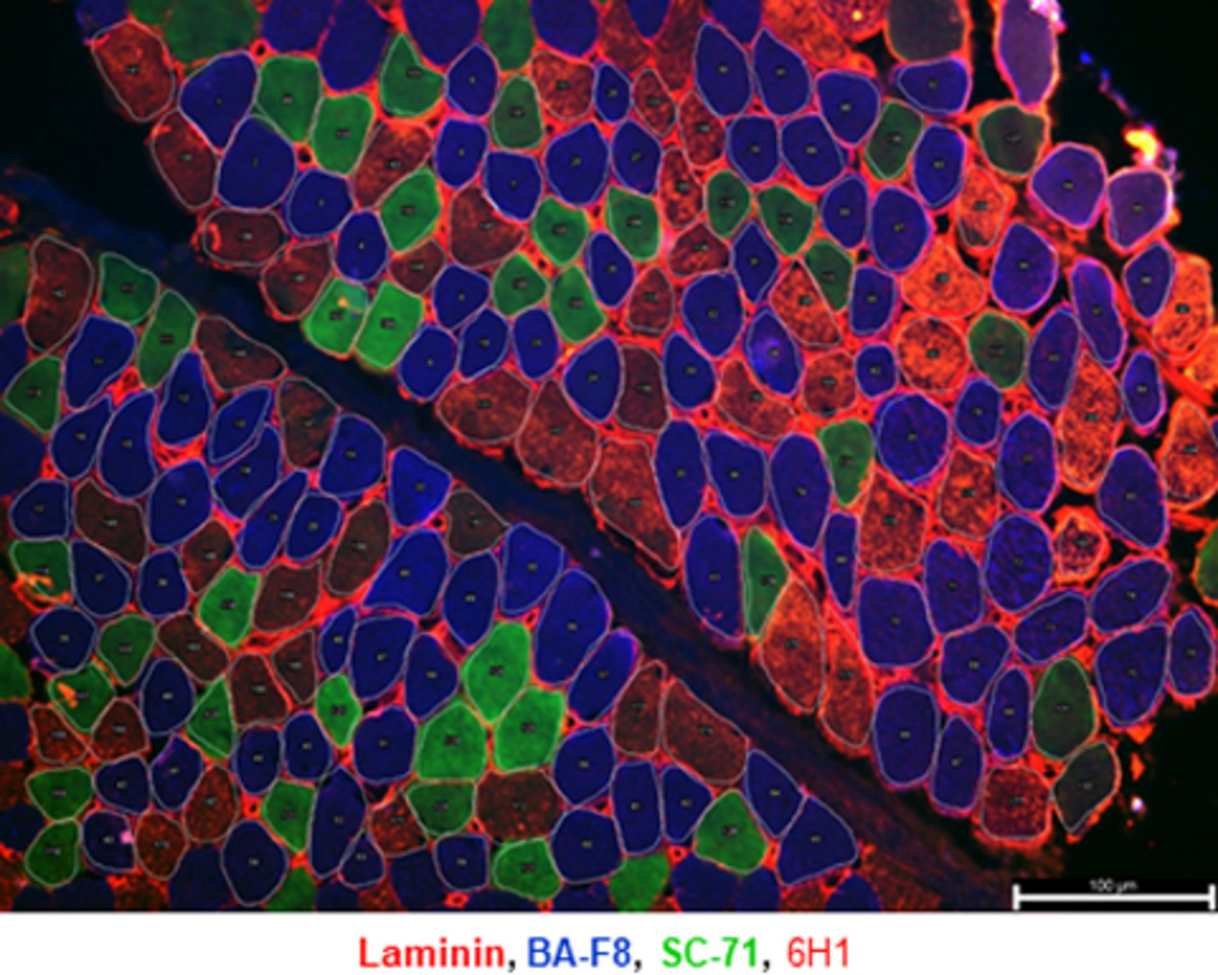
Representative example of a Medial Gastrocnemius section stained to highlight the different muscle fiber types. The section was stained with the different myosin heavy chain and laminin antibodies and the different fiber types were manually delineated using image J software. The type I fibers are stained in blue, the type IIa in green, the type IIx in red and laminin surrounding the fibers is depicted in red.

For each muscle, fiber proportion and size were assessed on a cryosection stained with an antibody cocktail specific to laminin, MHC-I, MHC-IIA, MHC-IIX (adapted from Bloemberg & Quadrilatero [30]). Each slide was air dried for 10 min at room temperature in a humid chamber and the muscle sections were delineated with a PAP pen (ab2601, Abcam). The samples were then blocked for one hour at room temperature with 10% normal goat serum in 1x phosphate buffer saline (7011044, Gibco® PBS). After removing the blocking solution, each section was incubated overnight at 4°C with the primary antibody cocktail against laminin (1:250, ab11575 Abcam), MHC type I (1:250, BA-F8), MHC type IIa (1:600, SC-71) and MHC type IIx (1:10, 6H1). The antibodies against the different MHC types were purchased from the Developmental Studies Hybridoma Bank (DSHB). Each section was then washed 3 times for 5 min in 1x PBS (7011044, Gibco® PBS) and incubated for 1 h at room temperature in the dark with the secondary antibodies Alexa fluor 680 (1:500, A-21076, Thermo Fisher Scientific), Alexa fluor 350 (1:500, A-21140, Thermo Fisher Scientific), Alexa fluor 488 (1:500, A-21121, Thermo Fisher Scientific), Alexa fluor 555 (1:500, A-21426, Thermo Fisher Scientific). After 3x5 min wash in 1x PBS (7011044, Thermo Fisher Scientific), the slides were mounted with ProLong® Gold antifade reagent (Molecular Probes, Thermofisher). Each section was then visualized under a fluorescence microscope (20x objective, Leica DMi8,) and pictures of each channel (DAPI, FITC, TRITC and CY5) were taken and saved as TIFF files. The stained section was subsequently stored at -20°C in the dark. To analyze the CSA and the proportion of the type I, type IIa, and type IIx fibers, the pictures of each channel were merged in LasX (Leica) and the area of each fiber as well as fiber type proportion were determined using Image J software by manually delineating each individual fiber (Fig 6). All cross-sectionally oriented fibers per field were counted, including at least 150 fibers per section.

Accuracy of the measurements was assessed by determining the intra-class correlation coefficients (with 95% confident interval) while comparing the results obtained by two different individuals analyzing the same muscle sections.

#### Satellite cell amount according to fiber type: Paired Box 7 (PAX7), MHC-I and DAPI immunostaining

To assess the amount of SCs and ensure their co-localization with myonuclei and fiber types, muscle sections were stained with antibodies against PAX7 for SCs, MHC-I, laminin, and DAPI for myonuclei according to a method adapted from Lindström et al (2009), Fry et al (2014), and Nederveen et al (2016) [31–33]. Each section was first air dried for 10 min at room temperature in a humid chamber and delineated with a PAP pen. After drying, the samples were fixed for 10 min in cold acetone at 4°C. After 3x5 min wash in 1x PBS (7011044, Thermo Fisher Scientific), each section was incubated for 1 h at room temperature in blocking buffer (10% normal goat serum in 1x PBS (7011044, Thermo Fisher Scientific) and then overnight at 4°C with a primary antibody cocktail against laminin (1:250, ab11575 Abcam), MHC-I (1:250, BA-F8 DSHB) and PAX7 (1:2, DSHB). The slides were subsequently washed 3x5min in 1x PBS (7011044, Thermo Fisher Scientific) and incubated for one hour at room temperature in the dark with the secondary antibody cocktail consisting in Alexa fluor 680 (1:500, A-21076, Thermo Fisher Scientific), Alexa fluor 594 (1:500, A-21145 Thermo Fisher Scientific) and Alexa fluor 488 (1:1000, A-21121 Thermo Fisher Scientific). Hereafter the sections were washed 3x5 min in 1x PBS (7011044, Thermo Fisher Scientific) and incubated for 1 min with DAPI (1:50, D1306, Thermo Fisher Scientific) and, after a final washing step of 3x5 min in 1x PBS (7011044, Thermo Fisher Scientific), mounted with ProLong® Gold antifade reagent (Molecular Probes, Thermofisher). Each section was then visualized on a fluorescence microscope (40x objective, Leica DMi8) and pictures of each channel were taken and saved as TIFF files. The stained slides were subsequently stored at -20°C in the dark. Quantification with ImageJ software was done after all channels were merged in LasX (Leica). SCs were identified as co-localization of PAX7 staining with DAPI and located in the sub-laminal region [19]. The amount of SCs, type I and II fibers was quantified on the entire muscle sample and for each muscle. The amount of SC/100 fibers for the type I and II fibers was calculated. Reproducibility of the measures was determined by calculating the intra-class correlation coefficients (with 95% confident interval) of the results obtained by three different individuals analyzing the same muscle sections.

#### Capillary amount according to fiber types: CD31 and MHC-I immunostaining

The slides were fixed, washed, and blocked in the same manner as for the SC staining. After blocking, the slides were incubated overnight at 4°C with a primary antibody-cocktail containing MHC-I (1:250, BA-F8 DSHB) and anti-CD31 (1:30, ab28364 Abcam). After incubation and 3x5 min washing step with 1x PBS (7011044, Thermo Fisher Scientific), the slides were incubated for 1 h in the dark with the corresponding secondary antibody Alexa fluor 488 (1:500, A-21121 Thermo Fisher Scientific) and Alexa fluor 555 (1:500, A-21428 Thermo Fisher Scientific). After incubation, the slides were again washed 3x5 min in 1x PBS (7011044, Thermo Fisher Scientific), followed by an overnight incubation at 4°C with the primary antibody anti-laminin (1:250, ab11575 Abcam) in the dark. After incubation and washing, the slides were incubated with the secondary antibody Alexa fluor 680 (1:500, A-21076, Thermo Fisher Scientific) for 1 h in the dark. The slides were again washed 3x5 min in 1x PBS (7011044, Thermo Fisher Scientific) and incubated for 1 min with DAPI (1:50, D1306, Thermo Fisher Scientific) and, after a final washing step of 3x5 min in 1x PBS (7011044, Thermo Fisher Scientific), mounted with ProLong® Gold antifade reagent (Molecular Probes, Thermofisher).

**Fig 7:**
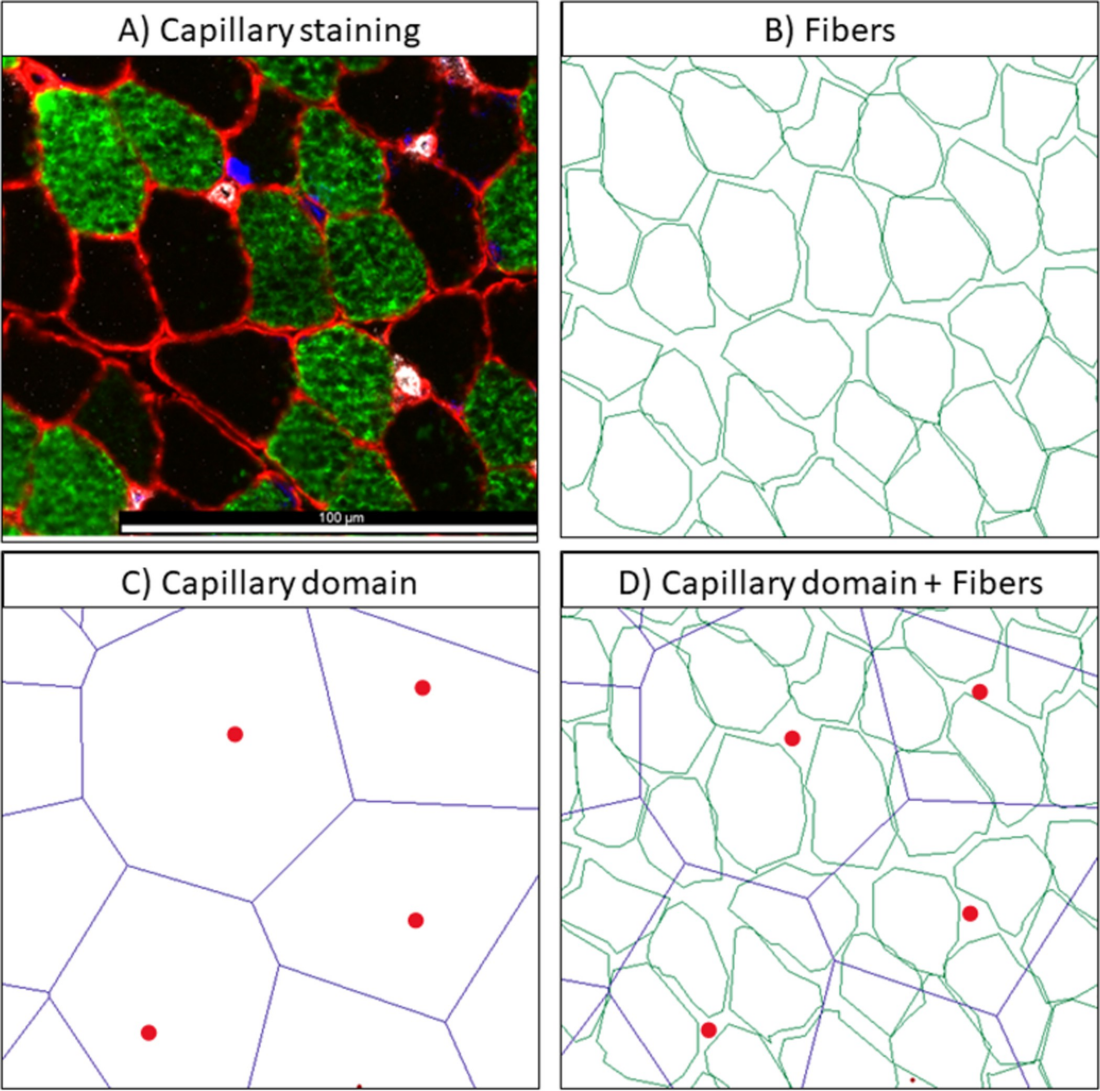
Overview of capillary staining and analysis. A) Representative example of a Medial Gastrocnemius section stained for capillary (CD31, white spots), type I (in green) and type II fiber (unstained, in black) and laminin (red); B) Muscle fibers from example A outlined and visualized in AnaTis application; C) and D) Capillary domain calculated with AnaTis application showing the capillaries as red dots and the capillary domain border being in equal distance between two neighboring capillaries. The overlap of the capillary domain and fibers is used to calculate different outputs.

The slides were visualized, and pictures were taken with a fluorescence microscope (20x objective, Leica DMi8). The fiber outlines and capillaries were traced in Btablet (BaLoH Software, www.baloh.nl, NL) converting the locations into coordinates. The acquired coordinates of the fiber outlines and capillary points were further analyzed with AnaTis (BaLoH Software, www.baloh.nl, NL). AnaTis calculates the capillary density as capillaries mm^-2^ tissue, capillary fiber density as capillaries mm^-2^ fiber, capillary-to-fiber ratio, the capillary domain defined as the region in equal distance between two neighboring capillaries, domains overlapping a fiber and the local capillary to fiber region (LCFR) which is the sum of the fractions of the capillary domains that are overlapping the muscle fiber (Fig 7). The capillary domain and LCFR take into account that the fiber can be supplied by capillaries that are not in direct contact with the fiber. These parameters give an estimation of the capillarization, hence the supply of oxygen, hormones and metabolites to the muscle fibers and removal of heat and waste products from the muscle [34,35].

Accuracy of the outcome measures was evaluated by determining the intra-class correlation coefficients (with 95% confident interval) of the results obtained by two different individuals analyzing the same muscle sections.

### Statistics

This paper mostly applies descriptive statistics reporting absolute and relative distribution expressed as absolute numbers and percentages. The inter-reliability of the different quantification methods is reported by the intra-class correlation coefficients (ICC) and 95% confident interval (CI) based on a two-way random effect model using an absolute agreement single measure. The guidelines of Koo et al were used to evaluate the level of reliability with values of ICC less than 0.5 representing a poor reliability, values between 0.5–0.75 showing a moderate reliability, values between 0.75 and 0.9 reflecting a good reliability and values greater than 0.9 corresponding to an excellent reliability [36]. Statistical analysis was performed with GraphPad Prism 9.2 for windows (GraphPad Software, San Diego, USA).

## Results

### Overview biopsy collection

Three to four biopsies per muscle were collected for each child of which two biopsies were handled for histology analysis. In total, 699 microbiopsies were collected in 88 children, from which 414 biopsies were used for histological analysis (Table 1). From these 414 microbiopsies, 288 were obtained in 56 children with CP (from MG and ST muscle in 46 children and solely from MG muscle in 10 children) and 126 were obtained in 32 TD children for both the MG and ST muscles.

**Table 1:** Population characteristics and number of biopsies collected per muscle type.

	Age (mean ± SD)	Gender (boy/girl)	GMFCS (I/II/III)	Muscle biopsy for histology: (MG/ST)
**CP**	5 yr 8 m (± 1 yr and 10 m)	33/23	19/23/14	163/125
**TD**	6 yr (± 1 yr and 10 m)	12/20	NA	63/63

CP: cerebral palsy, TD: typical developing, GMFCS: gross motor classification system, MG: Medial Gastrocnemius, ST: Semitendinosus. NA: not applicable.

In addition, in a subpopulation of the children with CP, repeated biopsies were taken in the MG 3 months (n = 7 children), 6 months (n = 8 children), 12 months (n = 12 children) after the first biopsy and in the ST 12 months (n = 12 children) after the first biopsy (Table 2).

**Table 2:** Population characteristics of CP children in whom repeated biopsies were taken and overview of the number of biopsies taken over time.

Time point repeated biopsy	MG	ST	Age (mean ± SD)	GMFCS (I/II/III)
**3 m after first biopsy**	7	NA	4 yr 8 m (± 2 yr and 1 m)	3/3/1
**6 m after first biopsy**	8	NA	4 yr 10 m (± 2 yr and 1 m)	3/3/2
**1 yr after first biopsy**	12	12	6 yr 5 m (± 2 yr)	3/6/3

MG: Medial Gastrocnemius, ST: Semitendinosus, GMFCS: gross motor classification system, NA: not applicable.

### Tolerance of the biopsy

**Fig 8:**
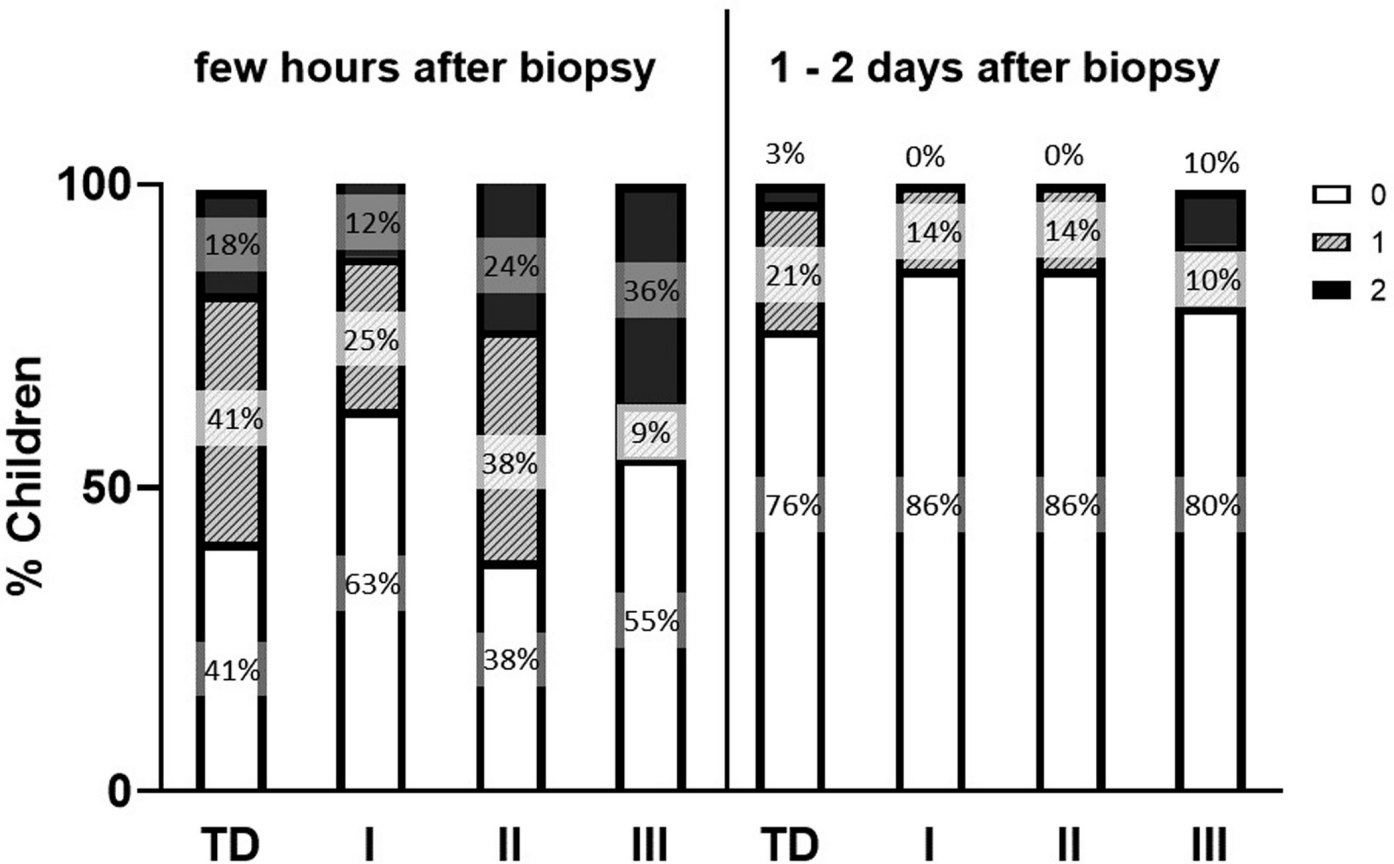
Pain assessment of TD children and children with CP according to GMFCS levels at day of biopsy and 1 to 2 days post biopsy. The pain score is based on a three-point score system: open bar (0): no or little pain, hatched bars (1): moderate pain, solid bars (2): severe to very severe pain.

At the day of biopsy, no pain was reported in 41% of the TD children and in 52% of the children with CP while moderate pain was described in 41% of the TD children and 24% of the children with CP. Severe pain was reported in 18% of the TD children and 24% of the children with CP, but in up to 36% of the CP children with GMFCS level III (Fig 8). One to 2 days after the biopsy, a great proportion of the children reported no (TD: 76%, CP: 84%) or moderate (TD: 21%, CP: 11%) pain and only 10% of the CP children with GMFCS level III and 3% of the TD children still reported severe pain (Fig 8). No statistical differences in pain score were observed between the different groups both at the day of biopsy and one to 2 days post biopsy. The pain score reported by the CP children in whom biopsies were taken under sedation with nitrous oxide and local anesthesia for repeated assessment (no pain: 40%, moderate pain: 40%, severe pain: 20%) was similar to the pain score reported by the CP children in whom biopsies were collected under general anesthesia. Pain medication was only very rarely taken specifically for the biopsy site. As the biopsy procedure was in most cases combined with another treatment, some children received pain medication for the pain of the additional procedure. Furthermore, no complications such as infection, neurovascular damages or wound healing impairments were reported.

### Hematoxylin and Eosin staining to analyze the quality and structure of the biopsy

In total, 132 biopsies were stained with H&E in children with CP (77 for the MG and 55 for the ST) and 62 biopsies in TD children (34 for the MG and 28 for the ST). In 4% of the MG biopsies and 11% of the ST biopsies of children with CP, there was no muscle material on H&E staining, while in TD children this was observed in 4% of the ST biopsies and this never happened for the MG biopsies (Table 3). The non-muscle material collected was identified as fat instead of muscle tissue.

**Table 3:** Overview and characteristics of the biopsies stained with Hematoxylin and Eosin.

	H&E staining (n)	No muscle tissue (%)	Fiber orientation		Freezing artefacts (%)	More than 150 fibers (%)
			Cross-sectional (%)	Longitudinal (%)		
**CP**	**132**	**7**	**86**	**8**	**28**	**60**
MG	77	4	87	9	33	68
ST	55	11	83	6	20	49
**TD**	**62**	**2**	**92**	**6**	**11**	**85**
MG	34	0	91	9	3	88
ST	28	4	93	4	7	71

CP: cerebral palsy, TD: typical developing, MG: Medial Gastrocnemius, ST: Semitendinosus, H&E: Hematoxylin and Eosin.

**Fig 9:**
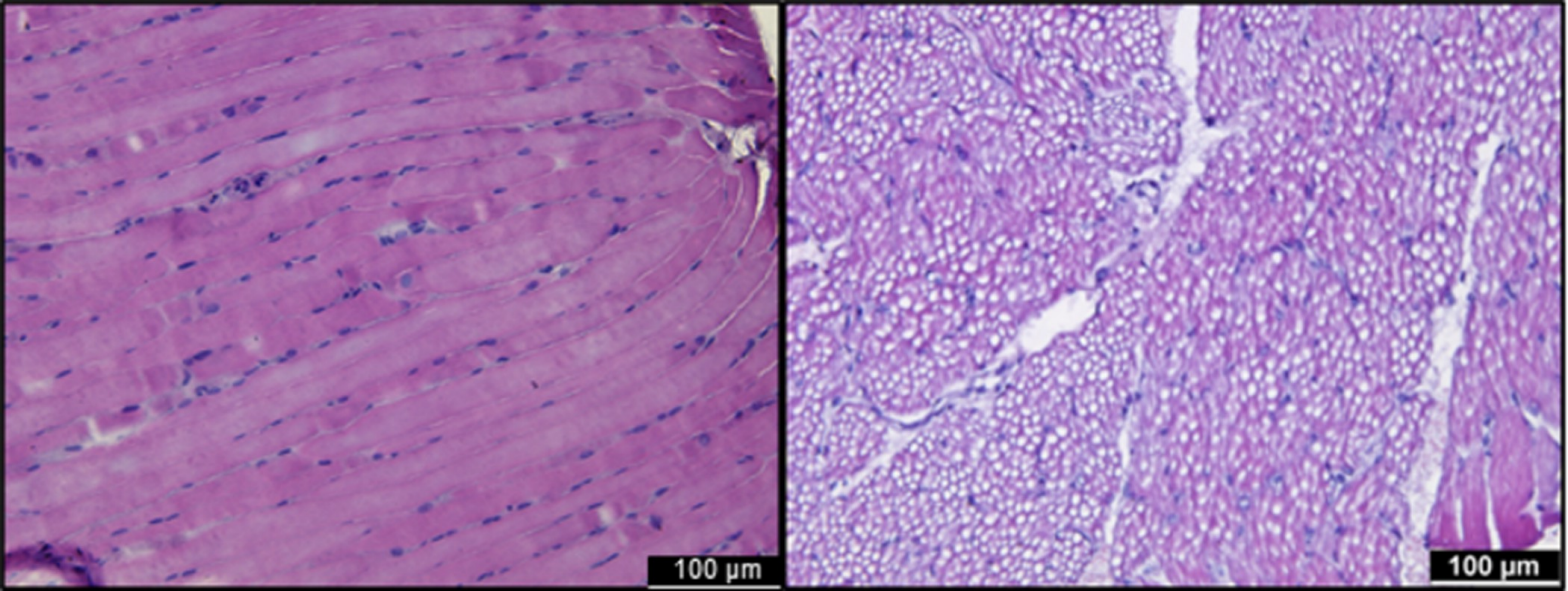
Representative examples of usable Hematoxylin and Eosin staining of the Medial Gastrocnemius section. Left panel: a section with a longitudinally oriented muscle fiber, right panel: a section with severe freezing artefacts (holes inside the tissue).

Longitudinal orientation of the muscle fibers was present in 8% of the biopsies in children with CP and 6% of the biopsies of TD children, but in most of the biopsies, a sufficient number of fibers with a cross-sectional orientation was available to address fiber size measurement (Table 3). A representative example of a MG section with muscle fibers that were longitudinally oriented is given in Fig 9 (left panel). Although not adequate to assess fiber CSA, longitudinal sections can be used to determine other parameters such as accumulation of nuclei as cluster along a muscle fiber [26].

In 28% of biopsies in children with CP and 11% of the biopsies in TD children, severe freezing artefacts were present on the whole surface of the muscle slide, making it difficult to find at least 150 fibers for further analysis as depicted in a representative example of a MG section in Fig 9 (right panel). In children with CP, 33% of the MG biopsies presented severe freezing artefacts compared to 20% for the ST biopsies. In TD children, severe freezing artefacts were present in 3% of the MG biopsies and 7% of the ST biopsies.

We therefore ended up with 68% of the MG biopsies and 49% of the ST biopsies in children with CP with no freezing artefact and at least 150 cross-sectionally oriented fibers. In TD children, 88% of the MG biopsies and 71% of the ST biopsies presented these criteria. However, since two biopsies from each muscle type were taken per child, 41 out of 46 children with CP and 20 children out of the 21 TD children had at least one usable MG biopsy while 21 out of 31 children with CP and 14 out of 18 TD children had at least one usable ST biopsy. In only 2 CP cases (4%), none of the biopsies taken for histology were suitable and this was due to severe freezing artefacts in both biopsies.

**Fig 10:**
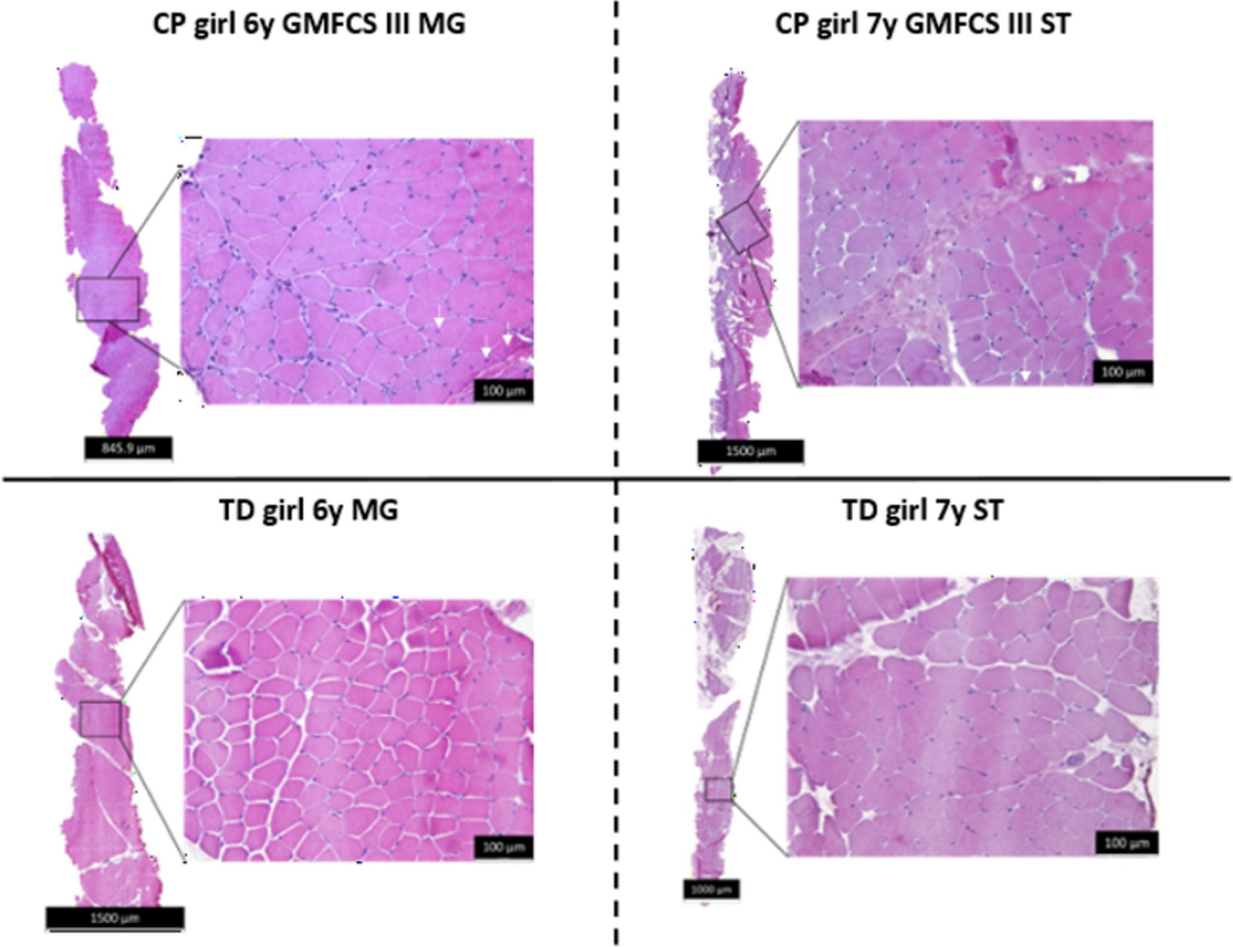
Representative examples of the Medial Gastrocnemius (left panels) and Semitendinosus (right panels) section stained with Hematoxylin and Eosin in girls with CP and GMFCS III (upper panels) and in age-matched TD (lower panels). The left part of each panel depicts the section of the entire muscle biopsy and on the right part of each panel the location from which the detailed image is generated. The small white arrows point to internal nuclei.

After evaluation of the quality of the biopsy transfer (fiber orientation) and the presence of freezing artefacts, several structural characteristics of the muscle such as fiber size and shape, excess of connective tissue, internal nuclei, inflammatory cells, and others could be examined [37–39]. Representative examples of an H&E staining from a MG (left panels) and ST (right panels) section are shown in Fig 10 for a child with CP and GMFCS III (upper panels) and an age- and gender matched TD child (lower panels). Fig 10 also shows the staining of the whole muscle biopsy on the left part of each panel and the location from which the detailed section shown on the right part is issued. Variability in fiber size and the presence of internal nuclei (small arrows) are visible in the MG and ST sections of the child with CP, while infiltration of potentially inflammatory cells (nuclei in extracellular matrix left side) is also seen in the MG section.

Quantitative evaluation of H&E stained section can be assessed by analyzing the normal and abnormal muscle fraction while using a point-grid system where each point-intercept is allocated to a specific event category (e.g. normal muscle, internal nuclei, inflammatory cell, necrotic fiber…) as previously described [37,40].

### Myosin heavy chain staining to determine the cross-sectional area and proportion of the different fiber types

MHC staining was performed on the MG microbiopsies of 35 children with CP and 27 children with TD and all sections were considered of good quality for further quantification (S1 Table). By contrast, ST staining was not successful in 9 out of 30 children with CP (30% failure) and 3 out of 19 TD children (16% failure) after minimal 2 attempts per biopsy of the 2 ST biopsies that were taken (S1 Table).

**Fig 11:**
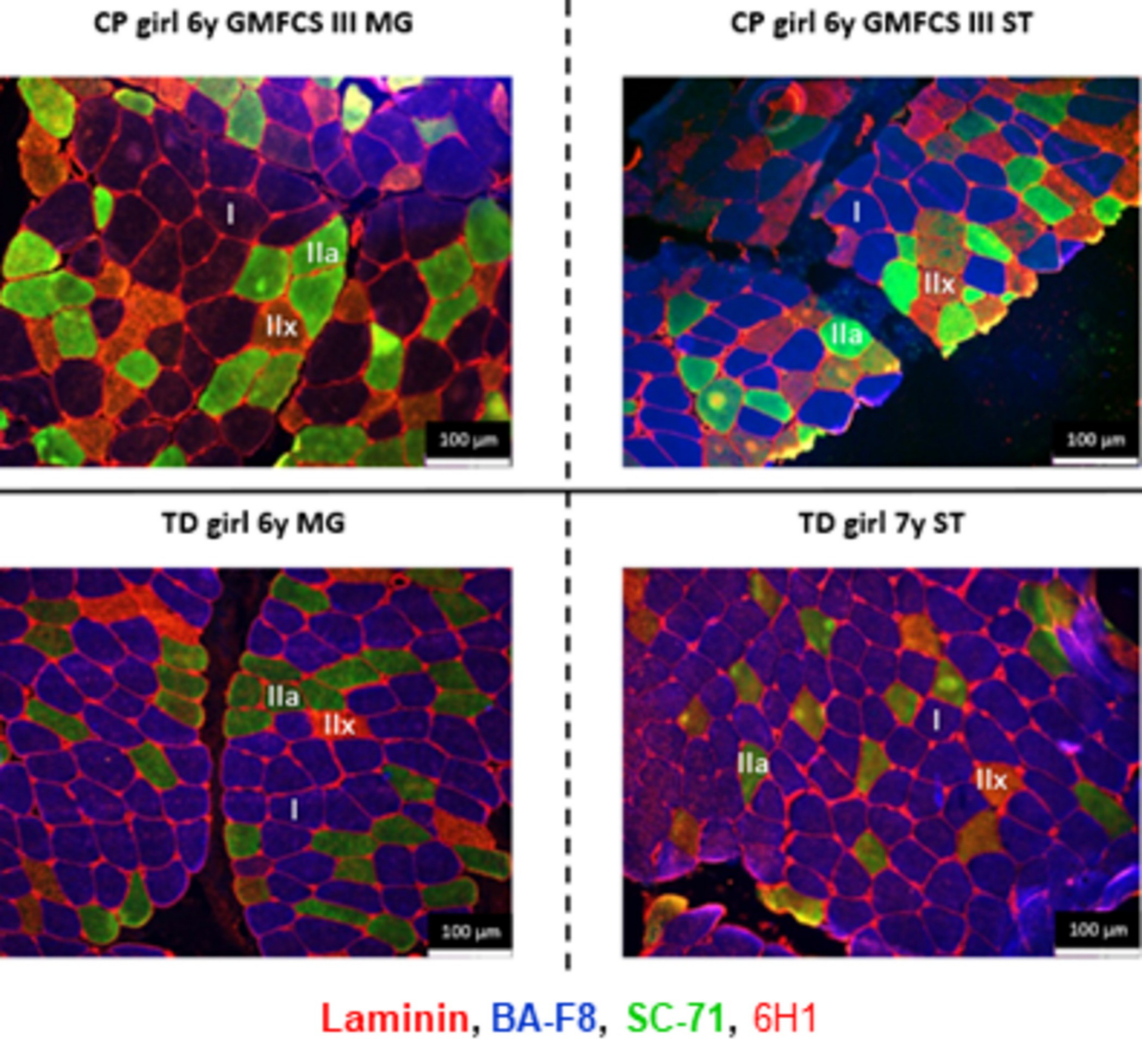
Representative examples of MHC staining of the Medial Gastrocnemius (left panels) and Semitendinosus (right panels) in girls with CP and GMFCS III (upper panels) and in age- and gender-matched TD children (bottom panels). Type I fibers are stained in blue, type IIa fibers in green, type IIx fibers in red and laminin surrounding the fibers is depicted in red.

Typical examples of a successful MHC staining for MG and ST sections are shown in Fig 11 for children with CP and aged-matched TD children. An example of an unsuccessful staining of the ST section is depicted in S1 Fig, where the individual staining for type I, IIa, IIx fibers and laminin as well as the merged image are provided. The picture of the merged images shows that it is impossible to distinguish the different fiber types, which is due to the fact that all fibers appear stained for type I, IIa and IIx as shown on the individual image while laminin is from low quality and not clearly visible (S1 Fig). Other examples of unsuccessful staining were due to the fact that all fibers stained in blue and/or green and/or red, which was always accompanied with a low-quality staining of laminin. Finally, representative examples of MHC staining performed at 2 time points in the same child with CP are provided in S2 Fig.

We were able to analyze 366 ± 210 fibers (CP) and 356 ± 162 fibers (TD) per child in the MG biopsies and 246 ± 82 fibers (CP) and 313 ± 150 fibers (TD) in the ST biopsies. A repeatability test for the CSA measures was performed independently by two different individuals who analyzed the CSA of all fibers from the same fields of view for each muscle type. For the MG, an ICC value of 0.976 (95% CI: 0.877–0.995) was obtained and for the ST, ICC reached 0.969 (95% CI: 0.856–0.993). There were no statistical differences in ICC values between the 2 muscle types or between CP and TD children.

### PAX7 immunofluorescence staining to quantify the amount of satellite cells

Staining of SC was performed in 34 children with CP (34 in MG, 20 in ST) and 27 TD children (27 in MG, 10 in ST). The majority of the SC staining was successful (S2 Table). In fact, for the children with CP, 11% (MG) and 30% (ST) of the SC stainings were unsuccessful while in TD children, this was the case in only 15% (MG) and 10% (ST).

**Fig 12:**
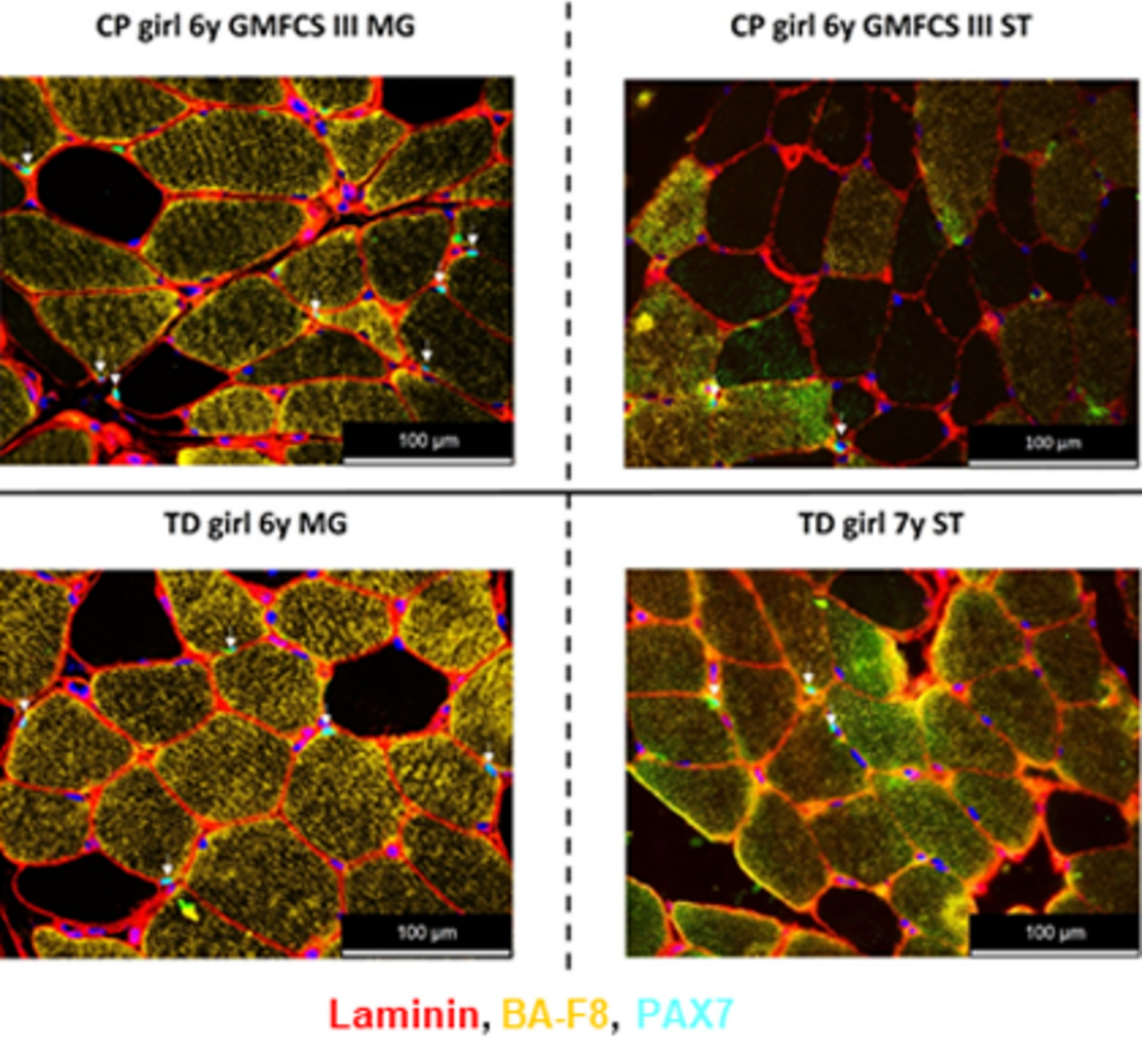
Representative examples of PAX7 staining of the Medial Gastrocnemius (left panels) and Semitendinosus (right panels) section from girls with CP and GMFCS III (upper panels) and an age-matched TD girl (lower panels). Type I fibers are stained in yellow; type II fibers are unstained and appear black, laminin is depicted in red, nuclei (DAPI) in blue and the SC are light blue spots indicated by arrows (co-localization of PAX7 in green and DAPI in blue).

Typical examples of PAX7 staining for MG and ST section are shown in Fig 12 for children with CP and TD children. We were able to quantify SC in 503 ± 261 fibers (CP) and 401 ± 221 fibers (TD) in the MG muscle and in 398 ± 119 fibers (CP) and 420 ± 165 fibers (TD) in the ST muscle.

The repeatability of the SC quantification was addressed independently by three individuals who measured the amount of SC on the same number of fields of view per muscle type. ICC values of 0.957 (95% CI: 0.869–0.985) and 0.931 (95% CI: 0.840–0.973) for the outcome SC/100 fibers were obtained for the MG and the ST, respectively with no statistical differences between the 2 muscle types or between CP and TD children.

### CD31 immunofluorescence staining to quantify the amount of capillaries

Staining of capillaries was performed in 33 children with CP (33 in MG, 14 in ST) and 20 TD children (20 in MG, 10 in ST) (S3 Table). Most of the muscle sections were stained successfully with CD31 with only 12% (MG) and 14% (ST) of the biopsies from children with CP and 10% of the ST biopsies from TD children resulting in an unsuccessful staining.

**Fig 13:**
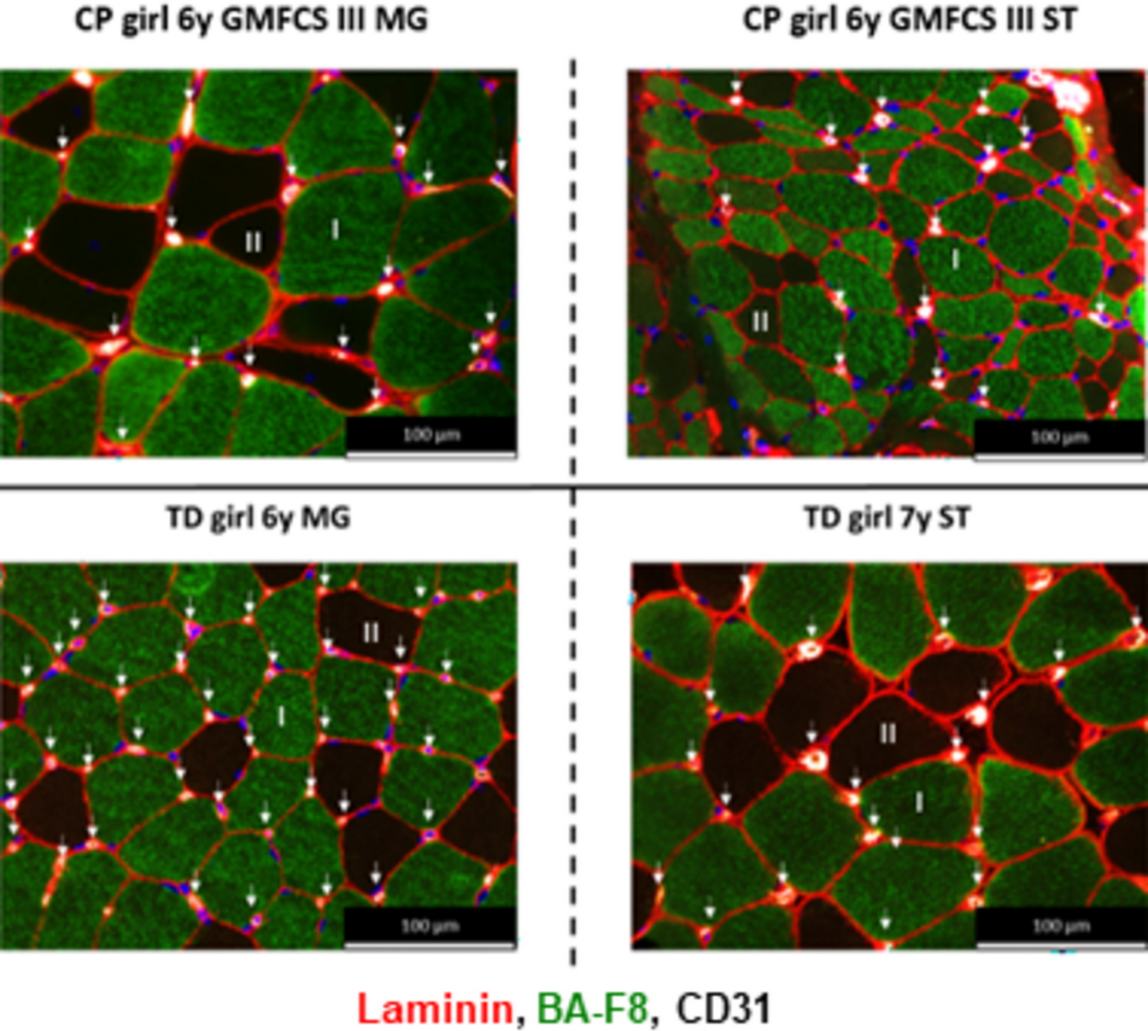
Representative examples of CD31 staining of the Medial Gastrocnemius (left panels) and Semitendinosus (right panels) section from girls with CP and GMFCS III (upper panels) and in age-matched TD girls (lower panels). Type I fibers are stained in green; type II fibers are unstained and appear black, laminin is depicted in red, nuclei in blue and the capillaries (CD31) are white dots shown by the arrows.

Representative examples of CD31 staining are depicted in Fig 13 for MG and ST sections in children with CP and TD children.

To address capillary amount, an average of 169 ± 110 fibers for the MG and 99 ± 18 fibers for the ST was counted in children with CP and 157 ± 85 fibers for the MG and 116 ± 35 fibers for the ST were analyzed for the TD children.

The repeatability of capillary measurement was addressed independently by two individuals who examined the number of capillaries on the same fields of view per muscle type. For capillary density, an ICC value of 0.947 (95% CI: 0.870–0.978) was obtained for the MG and an ICC value of 0.993 (95% CI: 0.742–0.999) was reached for the ST. For capillary to fiber ratio, ICC values were 0.957 (95% CI: 0.776–0.987) for MG and 9.92 (95% CI: 0.959–0.999 for ST. No statistical differences between the 2 muscle types or between CP and TD children were found for either outcome.

## Discussion

### Biopsy collection

Previous studies analyzing the muscle properties in children of CP mainly collected biopsies during surgery. For CP children, this occurred for example during tendon or hamstring lengthening surgery, while in TD children biopsies were collected during cruciate ligament reconstructive surgery or other surgery due to leg trauma or post-mortem [12–15,17–23,25].

The collection of muscle tissue using the open biopsy technique during surgery has the advantage that the collected biopsy has a high yield to analyze different biochemical and histochemical parameters. It is, however, restricted to the children undergoing surgery.

Furthermore, due to the invasiveness of surgery, it is difficult to obtain serial biopsies per patient to compare different muscles or to perform a longitudinal follow up study [27].

Only two studies have collected semi-open biopsies from the MG in patients with CP and healthy controls using the Bergström needle, one in adults [23] and for the other one, it is unclear whether these biopsies were collected in children or adults [16]. There are actually only a few studies reporting collection of Bergström biopsies in children in conditions other than CP [9,41–43]. These biopsies typically still require an incision through the skin and fascia and therefore are still painful and rather invasive (recovery time may be 6 days [44]) especially for collecting muscle biopsies in young children.

We believe that the ultrasound-guided percutaneous microbiopsy technique as used in the current study presents several advantages in comparison to the techniques commonly used to collect muscle biopsies in children with CP. As already reported, this technique is a good alternative that could help counterbalancing the limitations seen in previous studies [11,27,45,46]. The major advantage of the technique is the fact that it is less invasive and not associated with complications [11,27,45,46], this was also the case in our study. This has therefore allowed us to collect biopsies of two different muscles in a single sampling session. This is an important step forward as this will allow building a database of histological data from two muscle groups in TD children starting at the age of 3 yr, which will provide a valuable comparison with children to CP but also to children with other (neuro)muscular diseases. Having this histological database in TD children will likely help interpreting the heterogeneity or inconsistencies previously reported for microscopic muscle properties in the literature on children with CP. Next, we were able to show that sufficient muscle tissue can be collected using the percutaneous microbiopsy technique in order to perform different histological stainings and quantifications. Furthermore, we were able to collect up to four biopsies using the same puncture site. Besides that, we also showed the feasibility to collect successive biopsies from the same individual for longitudinal studies.

However, there are several points to consider when using this technique. First, it can be more challenging to obtain sufficient muscle fibers that are cross-sectionally oriented with this small muscle sample and this can result in the loss of the biopsy because only few muscle cells can be analyzed. To avoid losing data, it is highly recommended to take two biopsies per muscle for histological analysis in order to use the second biopsy in case the first one has to be excluded. According to our data with H&E staining, this would result in a success rate of 89% (CP) and 95% (TD) for the MG biopsies and 68% (CP) and 78% (TD) for the ST biopsies. Second, due to the biopsy size, the number of muscle slides for staining is limited, meaning that the number of assessments has to be well-thought in advance keeping also a back-up plan in mind in case a staining is unsuccessful. Despite the small size of the muscle sample, we were able to cut per muscle biopsy at least 10 slides with two sections per slide (20 sections in total with a thickness of 5 μm each), allowing the possibility to assess a range of analyses and also having some back-up samples. As earlier advised, in case a second biopsy is taken per muscle for histological assessment, this will offer 10 additional slides in case the first biopsy was also of good quality. Finally, although this study solely focused on the collection and analysis of lower limb muscles, we believe that this technique has a wider applicability and would also be suitable to collect biopsies from i.e. the upper limb muscles.

### Tolerance of the biopsy

In this study, we used the Wong-Baker faces pain scale [47] to assess the level of pain after the biopsy was collected. This pain scale is easy to use, requires minimal instructions, has been used in children aged 9 months to 18 years and was validated in the pediatric emergency department [29]. The current study revealed no differences in the reported pain level between TD children and children with CP and within the CP-subgroups. Half of the children reported little or no pain the day the biopsy was collected and this was even higher in CP children with GMFCS level I (55%) and III (63%). On the other hand, moderate pain was experienced by 41% of the TD children and 38% of the CP children with GMFCS level II and severe pain by 36% of the CP children with GMFCS level III. However, pain could be handled using an ice pack and only rarely required the use of a painkiller. These were prescribed but most parents found it was not necessary to use them. Finally, most of the children were pain free one or two days after the biopsies, indicating that pain was transient. In children with CP, the biopsies taken under anesthesia were always in combination with multilevel botulinum toxin injection and/or serial casting. These procedures can also cause pain in the treated muscles. Further, botulinum toxin injection or casting can cause temporary spasms shortly after the procedure in these children with spasticity. This may explain the higher percentage of children with more pain after the procedure in children with GMFCS III. Therefore, mandatory use of pain medication after this combined procedure, or at least using it early when presenting with -even mild- pain, might prevent or alleviate more symptoms. It should be noted that, depending on the surgery, some of the TD children received painkillers after the surgery to reduce pain and this might impact the pain assessment of these children. However, similar pain perception was found in other studies using percutaneous microbiopsies [11,28], suggesting that the impact of painkillers on pain assessment was likely limited. Previous studies have shown that the Bergström technique elicited larger perception of pain compared to the microbiopsy technique [11,27] with the latter inducing a pain level similar to a peripheral venous blood sampling [48]. Nevertheless, similar morphological analysis was achieved with both techniques [48]. In addition, these studies revealed that adult patients preferred the percutaneous microbiopsy technique above the Bergström and indicated that all participants would undergo a second biopsy using the microbiopsy technique if necessary [11,28]. In our study, many children with CP were willing to repeat the procedure of biopsy sampling and only 16% declined to participate for a follow-up biopsy. Moreover, in 7 out of these 8 children, we even collected biopsies at two different time points and did not face any complications and children experience the same level of pain as children in whom biopsies were collected at one time point. Interestingly, we were able to collect biopsies in the MG in young children with CP (age range: 3–9 yr) using local anesthesia and nitrous oxide as sedation instead of general anesthesia. Further, there were no reports on infection, neurovascular complications or wound healing alterations after the biopsy. These data show that the microbiopsy technique is safe and tolerable in patients, including young children in whom several biopsy specimens were taken from two different muscles.

### The quality and structure of the biopsy

Only a limited number of biopsies did not contain any muscle tissue and the amount was similar between TD children and children with CP and this concerns mainly the ST muscle. Previous studies did not report on the success rate of collecting muscle tissue using the microbiopsy technique. This is inherent to the technique and should be considered when designing a study as some samples might be lost.

Furthermore, to be able to analyze the muscle parameters of interest such as fiber CSA, number of SC and capillaries, the muscle fiber should be oriented cross-sectionally. However, a small proportion of the biopsies from the TD (6%) and CP (8%) children had fibers with a longitudinal orientation that were thus not usable for quantification of the chosen parameters. This is in line with the data of Agten et al (2018) where 4% of the biopsies had fibers not transversally orientated preventing fiber size analysis [28]. These authors also indicated that the amount of transverse-sectioned muscle fibers progressively increased from 153 to 352 fibers in consecutive biopsy samples during their study [28]. In our study, the number of fibers cross-sectionally oriented was in the same range as in the study from Agten et al (2018) for both the TD and CP children [28].

Additionally, 11% of the biopsies of TD children and 28% of the biopsies from children with CP showed severe freezing artefacts. Freezing artefacts are holes in the muscle tissue caused by the formation of ice crystals during the freezing step and are due to a high-water content or a slow freezing process. Freezing artifacts are unwanted since they can complicate or alter the evaluation of the muscle biopsy [49]. We were able to decrease the number of freezing artefacts by limiting the amount of OCT to the bare minimum, by decreasing the temperature of the isopentane in liquid nitrogen, and by prolonging the freezing time [49,50]. It is therefore highly recommended to pay attention to these steps when freezing the biopsy. In particular, the use of a minimal amount of OCT is key in this process. At the beginning of this study, excess OCT led to freezing artefacts, which were more frequent in the biopsies of the CP children (which we started taking before the biopsies in the TD children) compared to the TD children. But this has been considerably improved over time in our on-going study in CP children.

We were able to count an average of 503 ± 261 fibers and 401 ± 221 fibers in the MG muscle and an average of 398 ± 119 and 420 ± 165 fibers in the ST muscle of children with CP and TD children, respectively. Hayot et al (2005) counted a median of 144 fibers in a single section of a biopsy from the vastus laterals in adults and Agten et al (2018) reported 286 ± 217 cross-sectional fibers in the erector spinae muscle and 210 ± 160 fibers in the lumbar multifidus muscle in adults [27,28]. The higher number of fibers seen in our biopsy sample is likely explained by the fact that the fiber CSA in children is smaller compared to that of adult muscle samples, also keeping in mind that the muscle types were different.

We considered a biopsy as usable for histological analysis when the biopsy had at least 150 cross-sectionally oriented fibers with little or no freezing artefacts. We found a different success rate in the biopsies depending on the muscle group sampled and on whether the child was a TD child or had CP. Biopsies taken in children with CP were less successful (60%) compared to the biopsies taken in children in TD (85%). Moreover, the success rate was lower for the ST (CP: 49% and TD: 71%) compared to the MG (CP: 68% and TD: 88%). As explained earlier, we had to face troubles with the OCT at the beginning of the study and this resulted in severe freezing artefacts notably in CP biopsies that were first collected in this study. This might explain the different success rate between CP and TD children. Another reason for these differences is related to the amount of muscle tissue collected compared to other non-muscle material present in the biopsy so that muscle material was not sufficient to provide enough cross-sectionally oriented fibers. These results on the quality of the biopsies show that it is advised to collect at least two biopsies for histology per muscle per patient.

Taking together, these data underline that, while in the current study some biopsies were lost because the muscle fibers were not appropriately oriented for the chosen analysis or due to freezing artefact, the remaining biopsies provided sufficient numbers of fibers for analysis, which is in line with previous studies using the microbiopsy technique for other muscle types in adults [27,28]. By gaining more experience in the techniques, we noticed an improvement in quality of the muscle biopsies over time. Such learning curve was also reported by Agten et al (2018) [28].

### MHC staining to determine the cross-sectional area and proportion of the different fibers

At the moment there are multiple studies reporting the CSA and fiber type distribution in children with CP in different muscle types [2,7,17,18,20,24,51,52]. However, there are still no data on the CSA and fiber type proportion in very young children with CP and TD children.

The staining described in our methodology was successful for the MG muscle biopsies from both children with CP and TD children, contrary to the ST muscle, particularly in children with CP. So far, we have no explanation why the staining was less successful in the ST compared to the MG. To our knowledge, there are no previous studies reporting similar issues. However, we do hypothesize that the problem is not related to the microbiopsy technique but is rather muscle related and more particularly in CP. The problem is also not related to technical handling during the staining step since these unsuccessful stainings were processed in parallel with other successfully stained muscle biopsies. This issue has to be taken into account when analyzing different muscles in CP children. However, H&E staining can still be used as an alternative to assess fiber dimensions in unsuccessful MHC staining, whereby the results can be depicted as histograms and the frequency of a given fiber can be reported according to serial range of fiber size. Regarding the amount of fibers analyzed to assess fiber dimensions, Nederveen et al. (2020) showed that a minimum of 150 fibers is needed for an accurate representation (CV < 5%) of the muscle CSA and fiber type proportion in healthy adults [53]. Taking these results into account, we aimed to count at least 150 fibers per muscle and per child in the present study and we also showed that the quantification protocol between different analysts yielded reproducible and accurate results. This current study therefore shows that it is feasible to analyze the CSA and fiber type proportion in very young children including children with CP using muscle sample collected with the microbiopsy technique.

### PAX7 immunofluorescence staining to quantify the number of satellite cells

We showed a relative high success rate with this staining for the MG and ST in children with CP and TD. SCs are not equally distributed in the muscle and Mackey et al (2009) showed that at least 50 type I fibers and 75 type II fibers need to be counted for the vastus lateralis in healthy adult muscles to ensure reliable estimation [54]. However, they suggest to count the entire muscle section to increase reliability [54]. We therefore opted to quantify SCs in the entire biopsy section to avoid selection bias and to provide accurate estimation of the number of SCs. We were far beyond the amount of 50 type I and 75 type II fibers recommended by Mackey et al [54] as an average of 503 ± 261 fibers in CP and 401 ± 221 fibers in TD were assessed in the MG muscle and an average of 398 ± 119 fibers in CP and 420 ± 165 fibers in TD were quantified in the ST muscle. Our quantification technique showed to be reproducible between different analysts. Other studies analyzing SC counts in healthy subjects reported an average quantification of 240 ± 23 for type I and 182 ± 11 for type II fibers in muscle biopsies from different muscles in children undergoing surgery (<18 years) and from the vastus lateralis in healthy adults [55]. Up to 569 ± 81 fibers have also been analyzed in biopsies of the vastus lateralis in healthy adults to determine SC counts [56]. Only one study has analyzed the amount of SC in the gracilis muscle of CP children with PAX7 staining as we did in the current study [19]. The authors reported the quantification of an average of 175 fields (40x objective, field size: 237 x 178 μm) per section to assess SC counts. The latter is, however, not possible with the microbiopsy technique where an average of 13 ± 5 fields (40x objective, field size: 308 x 231 μm) can only be counted per section. Yet, we were able to analyze the recommended number of fibers, and even more to estimate the number of SC. This indicates that the small microbiopsy size does not preclude an accurate estimation of the SC counts, but it is advised to analyze the entire muscle section if possible/available to increase reliability.

### CD31 immunofluorescence staining to quantify the number of capillaries

Our study showed a high success rate with the staining of capillaries in children with CP and TD children for both the MG and the ST muscle. Neverdeen et al (2020) showed that there is a homogeneous distribution of markers for muscle capillarization in the vastus lateralis in healthy adults. However, they suggested to analyze a minimum of 50 fibers and/or 30% of the total sample to determine the amount of capillaries within one biopsy notably for the capillary to fiber ratio and capillary to fiber perimeter exchange index [53]. We quantified 100 fibers on average per section, which is twice the recommended amount for acceptable quantification of capillarization. In addition, the amount of capillaries analyzed per child (34–381) was in the same range as previously reported to assess capillary density, capillary to fiber ratio, and capillary domain size using the same technique as in the current study [35]. One study analyzing the vastus lateralis of healthy older men quantified 30 type I and type II fibers [57,58], and another study quantified 50 fibers in the vastus lateralis of healthy adults [33]. This shows that we were able to count sufficient numbers of fibers to obtain a reliable result. To quantify the number of capillaries, we used the Btablet and AnaTis applications (from BaLoH Software, www.baloh.nl) and were able to show that this method provides accurate reproducibility with repeated measurements between different analysts. These data indicate the feasibility to analyze capillarization in biopsies from the MG and ST in children with CP and TD collected using the percutaneous microbiopsy technique.

## Conclusions

This study shows for the first time the feasibility and safety of using the fine-needle percutaneous microbiopsy technique to collect skeletal muscle biopsies from the MG and the ST muscle in young children with CP and in young TD children for histological assessments. Severe pain reported after the biopsy collection (TD: 18%, CP: 24%) and two days later (TD and CP: 3%) should be handled with painkiller especially when the biopsy is combined with other procedures which can also contribute to pain. This study furthermore showed that sufficient amounts of good quality muscle tissue can be collected using this minimally invasive technique and that different (immuno-) staining’s can be performed on the same muscle sample using multiple muscle sections. We have shown that this technique is suitable for the assessment of muscle structure, muscle fiber dimension and the proportion of different muscle fiber types, besides quantification of satellite cell and capillary number. Nevertheless, as for the other biopsy techniques, it is recommended to count sufficient muscle tissue to ensure reliability of the obtained measurements.

## Supporting information

S1 Fig Typical example of an unsuccessful MHC staining from a semitendinosus section. Individual staining for A) Type I, B) Type IIa, C) Type IIx and D) Laminin is depicted as well as E) The merged image of the 4 staining’s.
(PDF)

S2 Fig Representative examples of MHC staining from biopsies collected at two different time points in the same child with CP. The child is a boy, GMFCS level II. Type I fibers are stained in blue, type IIa fibers in green, type IIx fibers in red and laminin surrounding the fibers is depicted in red. At time point 1, CP child was 7.8-year-old. Time point 2: 14 months later.
(PDF)

S1 Table Number of successful and unsuccessful myosin heavy chain staining for the medial gastrocnemius and semitendinosus biopsies in children with cerebral palsy and typical developing children. (DOCX)

S2 Table Number of successful and unsuccessful satellite cell staining in the medial gastrocnemius and semitendinosus section of children with cerebral palsy and typical developing children. (DOCX)

S3 Table Number of successful and unsuccessful capillary staining in the medial gastrocnemius and semitendinosus section of children with cerebral palsy and typical developing children. (DOCX)
