# Supplementary material for: Fine-needle percutaneous muscle microbiopsy technique as a feasible tool to address histological analysis in young children with cerebral palsy and age-matched typically developing children
Source: PLoS One. 2023 Nov 22;18(11):e0294395. doi: 10.1371/journal.pone.0294395 (PMC10664906; doi:10.1371/journal.pone.0294395)

S1 Figure. Typical example of an unsuccessful MHC staining from a Semitendinosus section. Individual staining for A) Type I, B) Type IIa, C) Type IIx and D) Laminin is depicted as well as E) The merged image of the 4 staining's

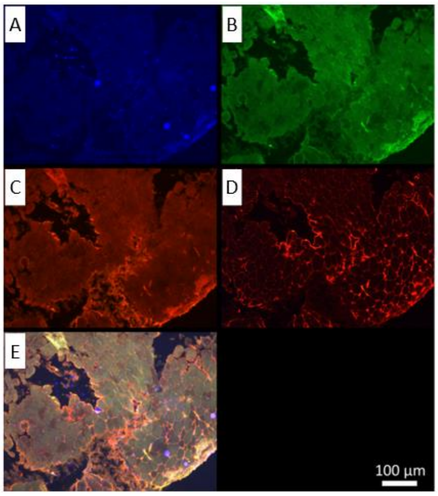

Supplement: S1 Fig — Individual staining for A) Type I, B) Type IIa, C) Type IIx and D) Laminin is depicted as well as E) The merged image of the 4 staining’s. (PDF) [file pone.0294395.s001.pdf]
