# Supplementary material for: Fine-needle percutaneous muscle microbiopsy technique as a feasible tool to address histological analysis in young children with cerebral palsy and age-matched typically developing children
Source: PLoS One. 2023 Nov 22;18(11):e0294395. doi: 10.1371/journal.pone.0294395 (PMC10664906; doi:10.1371/journal.pone.0294395)

S2 Fig. Representative examples of MHC staining from biopsies collected at two different time points in the same child with CP. The child is a boy, GMFCS level II. Type I fibers are stained in blue, type IIa fibers in green, type IIx fibers in red and laminin surrounding the fibers is depicted in red. At time point 1, CP child was 7.8-year-old. Time point 2: 14 months later

Time point 1

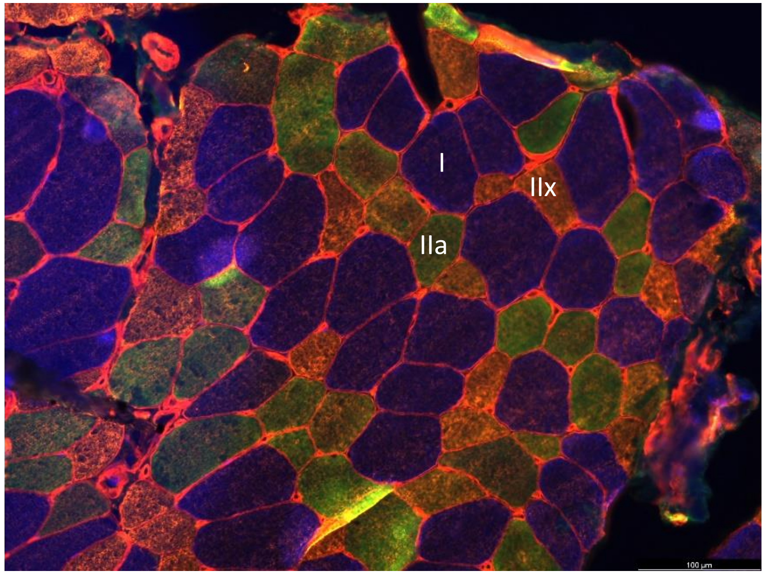

Time point 2

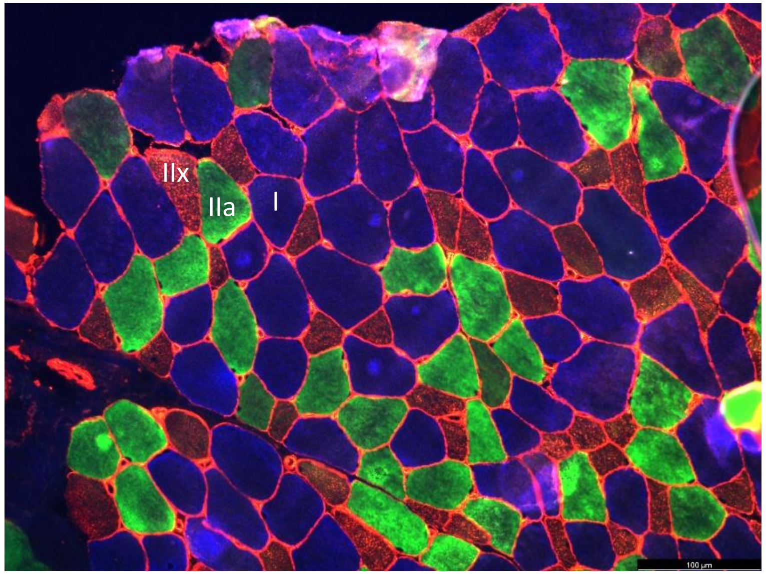

Supplement: S2 Fig — The child is a boy, GMFCS level II. Type I fibers are stained in blue, type IIa fibers in green, type IIx fibers in red and laminin surrounding the fibers is depicted in red. At time point 1, CP child was 7.8-year-old. Time point 2: 14 months later. (PDF) [file pone.0294395.s002.pdf]
