# Supplementary material for: Fine-needle percutaneous muscle microbiopsy technique as a feasible tool to address histological analysis in young children with cerebral palsy and age-matched typically developing children
Source: PLoS One. 2023 Nov 22;18(11):e0294395. doi: 10.1371/journal.pone.0294395 (PMC10664906; doi:10.1371/journal.pone.0294395)
Supplement: S1 Table — (DOCX) [file pone.0294395.s003.docx]

**S1 Table. Number of successful and unsuccessful myosin heavy chain staining for the medial gastrocnemius and semitendinosus biopsies in children with cerebral palsy and typical developing children.**

|  | CP | | TD | |
| --- | --- | --- | --- | --- |
|  | MG | ST | MG | ST |
| Biopsies with successful MHC staining | 35 | 21 | 27 | 16 |
| Biopsies with unsuccessful MHC staining | 0 | 9 | 0 | 3 |

CP: cerebral palsy, TD: typical developing, MG: Medial Gastrocnemius, ST: Semitendinosus, MHC: myosin heavy chain
