# Supplementary material for: Fine-needle percutaneous muscle microbiopsy technique as a feasible tool to address histological analysis in young children with cerebral palsy and age-matched typically developing children
Source: PLoS One. 2023 Nov 22;18(11):e0294395. doi: 10.1371/journal.pone.0294395 (PMC10664906; doi:10.1371/journal.pone.0294395)
Supplement: S2 Table — (DOCX) [file pone.0294395.s004.docx]

**S2 Table. Number of successful and unsuccessful satellite cell staining in the medial gastrocnemius and semitendinosus section of children with cerebral palsy and typical developing children.**

|  | CP | | TD | |
| --- | --- | --- | --- | --- |
|  | MG | ST | MG | ST |
| Biopsies with successful PAX7 staining | 30 | 14 | 23 | 9 |
| Biopsies with unsuccessful PAX7 staining | 4 | 6 | 4 | 1 |

CP: cerebral palsy, TD: typical developing, MG: Medial Gastrocnemius, ST: Semitendinosus, PAX7: Paired Box 7
